# Fixel-based analysis of the preterm brain: Disentangling bundle-specific white matter microstructural and macrostructural changes in relation to clinical risk factors

**DOI:** 10.1016/j.nicl.2019.101820

**Published:** 2019-04-10

**Authors:** Diliana Pecheva, J-Donald Tournier, Maximilian Pietsch, Daan Christiaens, Dafnis Batalle, Daniel C. Alexander, Joseph V. Hajnal, A. David Edwards, Hui Zhang, Serena J. Counsell

**Affiliations:** a Centre for the Developing Brain, School of Biomedical Engineering & Imaging Sciences, King'’s College London, UK; b Sackler Institute for Translational Neurodevelopment, Department of Forensic and Neurodelopmental Science, Institute of Psychiatry, Psychology & Neuroscience, King'’s College London, UK; c Department of Computer Science and Centre for Medical Image Computing, University College London, UK

**Keywords:** Brain, Preterm, Diffusion MRI, Fixel-based analysis, dMRI, Diffusion MRI, FBA, Fixel-based analysis, FC, Fibre cross-section, FD, Fibre density, FDC, Fibre density x fibre cross-section, GA, Gestational age, PMA, Post-menstrual age, TPN, Total parenteral nutrition

## Abstract

Diffusion MRI (dMRI) studies using the tensor model have identified abnormal white matter development associated with perinatal risk factors in preterm infants studied at term equivalent age (TEA). However, this model is an oversimplification of the underlying neuroanatomy. Fixel-based analysis (FBA) is a novel quantitative framework, which identifies microstructural and macrostructural changes in individual fibre populations within voxels containing crossing fibres. The aim of this study was to apply FBA to investigate the relationship between fixel-based measures of apparent fibre density (FD), fibre bundle cross-section (FC), and fibre density and cross-section (FDC) and perinatal risk factors in preterm infants at TEA. We studied 50 infants (28 male) born at 24.0–32.9 (median 30.4) weeks gestational age (GA) and imaged at 38.6–47.1 (median 42.1) weeks postmenstrual age (PMA). dMRI data were acquired in non-collinear directions with b-value 2500 s/mm^2^ on a 3 Tesla system sited on the neonatal intensive care unit. FBA was performed to assess the relationship between FD, FC, FDC and PMA at scan, GA at birth, days on mechanical ventilation, days on total parenteral nutrition (TPN), birthweight z-score, and sex. FBA reveals fibre population-specific alterations in FD, FC and FDC associated with clinical risk factors. FD was positively correlated with GA at birth and was negatively correlated with number of days requiring ventilation. FC was positively correlated with GA at birth, birthweight z-scores and was higher in males. FC was negatively correlated with number of days on ventilation and days on TPN. FDC was positively correlated with GA at birth and birthweight z-scores, negatively correlated with days on ventilation and days on TPN and higher in males. We demonstrate that these relationships are fibre-specific even within regions of crossing fibres. These results show that aberrant white matter development involves both microstructural changes and macrostructural alterations.

## Highlights

• Fixel-based analysis of effects of risk factors on preterm brain development
• Differentiates micro- and macrostructural changes in individual fibre bundles
• Fibre density and cross-section decrease with low gestation and longer ventilation.
• A number of clinical risk factors are related to lower fibre cross-section.

## Graphical abstract

**Figure:**
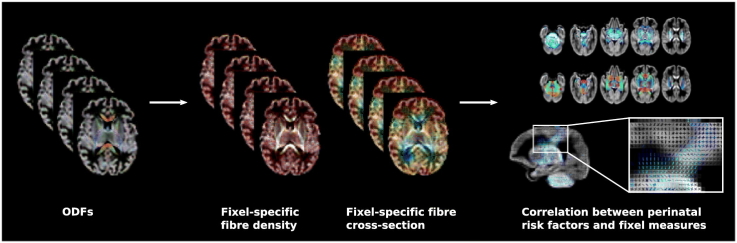

## 1 Introduction

Preterm birth affects 1 in 10 births worldwide (Howson et al., 2013), and is a leading cause of infant mortality and morbidity (Blencowe et al., 2012). The adverse consequences of prematurity encompass a wide range of impairments including cognitive, behavioural (Bayless and Stevenson, 2007; Bhutta et al., 2002; Delobel-Ayoub et al., 2009; Joseph et al., 2016; Marlow et al., 2005), motor (Marlow et al., 2007; Williams et al., 2010; Wood et al., 2005) and language delays (Allin et al., 2008; Guarini et al., 2009; Wolke and Meyer, 1999; Wolke et al., 2008). Moreover, perinatal risk factors such as the need for respiratory support, parenteral nutrition, growth restriction and infection are common in the preterm population and are associated with an increased risk of brain injury and poor outcome (Bernstein et al., 2000; Brouwer et al., 2017; Kady and Gardosi, 2004; Kobaly et al., 2008; Lin and Stoll, 2006; Lodha et al., 2014; Padilla et al., 2014; Short et al., 2003; Stephens et al., 2009; Vohr et al., 2000).

The cerebral white matter is particularly vulnerable to injury in preterm infants (Volpe, 2003, Volpe, 2009a, Volpe, 2009b). Quantitative magnetic resonance imaging (MRI) approaches, such as diffusion MRI (dMRI), have been used extensively to examine white matter development and injury in this population. Measures derived from the diffusion tensor model are sensitive to developmental changes and alterations in white matter related to prematurity and perinatal risk factors (Anjari et al., 2009; Ball et al., 2010; Chau et al., 2009; Zwicker et al., 2013). Nonetheless, this model is an over simplification of the underlying anatomy. White matter voxels contain crossing fibres, with up to 90% of voxels containing multiple distinct fibre populations in the adult brain (Behrens et al., 2007; Jeurissen et al., 2013). However, the diffusion tensor expresses a single principal direction and is unable to characterise more than one fibre orientation. This is especially important for tensor-based tractography methods which may fail in regions where measured fractional anisotropy (FA) is low, including regions of complex fibre architecture and branching structures such as the lateral projections of the corpus callosum (Tournier et al., 2011). One approach to overcome this limitation is constrained spherical deconvolution (CSD) (Tournier et al., 2007; Tournier et al., 2004) which can resolve multiple fibre populations within a voxel. We have previously applied CSD to preterm neonatal populations to study white matter tracts with complex configurations that are not possible to resolve with diffusion tensor imaging (DTI) (Pieterman et al., 2017; Salvan et al., 2017). However, these studies reported voxel-averaged DTI measures in their assessments of white matter microstructure and did not incorporate macrostructural measures.

Volumetric tissue changes can be assessed using tensor-based morphometry (TBM), based on the spatial transformations derived from image registration of each subject to a common template. The determinant of the spatial derivative matrix (the Jacobian) describes the local expansion or contraction of each subject's image with respect to the template. However, this discards information regarding the spatial direction of volume change. With respect to white matter, a change perpendicular to the fibre bundle is more likely to reflect a functionally relevant change than a change in tract length. Zhang et al. (2009) introduced a TBM-based approach which quantified the volumetric change in the plane perpendicular to the main fibre bundle orientation. This method provided a more meaningful and sensitive measure of tissue morphology, nonetheless, it was still limited by the tensor model.

Fixel-based analysis (FBA) is a novel framework which provides quantitative measures of macro- and microstructure associated with a single fibre population, even in voxels containing crossing fibres (Raffelt et al., 2017). In FBA, a higher-order diffusion model is used to compute fibre orientation density functions (ODF) from which individual fibre populations are extracted. Individual fibre bundle elements within a voxel are referred to as fixels. FBA allows the group-wise comparison of fixel-specific apparent fibre density, morphometric change in fibre bundle cross-section, and the combined measure of fibre density and bundle cross-section.

Apparent fibre density (FD) estimates the volume of the restricted intra-axonal compartment for a particular fibre population direction within a voxel. A reduced FD may be due to fewer axons in that fibre population or due to smaller axons. This makes FD sensitive to changes in tissue microstructure. For the assessment of morphometric group differences, fibre cross-section (FC) captures fixel-specific changes in fibre-bundle cross-sectional area. FC is based on the transformations derived from ODF registration, analogous to the tensor-based method from Zhang et al. (2009). Decreases in FC reflect a reduction in the spatial extent occupied by the tract that may be due to impaired axonal growth or due to atrophy following an insult.

Fibre density and cross-section (FDC) is the fibre density weighted by the change in the cross-sectional extent of the tract. Assuming that the total FD across a fibre bundle is a measure of its capacity to relay information, FDC allows comparisons of this capacity, irrespective of morphometry. Recently, Pannek et al. (2018) demonstrated the feasibility of applying FBA to study white matter during the perinatal period for a preterm cohort, assessing alterations in white matter related to prematurity, age at scan and brain abnormalities. However, the study did not evaluate the impact of clinical risk factors on white matter.

The aim of this study was to investigate the relationship between fixel-based measures and a number of clinical risk factors including degree of prematurity, the number of days on mechanical ventilation, sex, the number of days on total parenteral nutrition (TPN), and birthweight. We hypothesise that greater exposure to these risk factors is associated with lower FD and FC. We expect FBA to provide new insights into whether risk factors affect whole-brain white matter or whether the effects are localised. Furthermore, in crossing fibre regions FBA will help to discern whether individual tracts are affected or whether these effects are regional, traversing multiple white matter tracts.

## 2 Methods

### 2.1 Subjects

We studied 50 infants (28 male) born at 24.0–32.9 (median 30.4) weeks gestational age (GA) and imaged at 38.6–47.1 (median 42.1) weeks postmenstrual age (PMA). Infants were recruited as part of the Evaluation of Preterm Imaging (*E*-Prime) study of preterm brain development. Written parental consent was obtained prior to imaging. T1- and T2-weighted MR images were assessed for the presence of focal brain injury. The perinatal characteristics of the infants were obtained from clinical records at the time of MRI. A two-sample Kolomorov-Smirnoff test was applied to test for differences between male and female subjects in perinatal clinical characteristics. We found no significant sex differences in any of the perinatal characteristics studied.

### 2.2 MR data acquisition

3D MPRAGE (Magnetization Prepared Rapid Acquisition Gradient Echo, TR 17 ms; TE 4.6 ms; flip angle 13°; slice thickness 0.8 mm; in plane resolution 0.82 × 0.82 mm), T2-weighted turbo spin echo (TR 8670 ms; TE 160 ms; flip angle 90°; slice thickness 2 mm; in plane resolution 0.86 × 0.86 mm) and high angular resolution diffusion imaging (HARDI) data (64 non-collinear directions, b = 2500 s/mm2 and 4 non-diffusion-weighted images, TR = 9000 ms, TE = 62 ms, voxel size, 2 mm isotropic, SENSE factor of 2) were acquired on a Philips 3 Tesla (Philips Medical Systems, Best, The Netherlands) system sited on the neonatal intensive care unit using an eight-channel phased array head coil. All examinations were supervised by a pediatrician experienced in MR imaging. Pulse oximetry, temperature and electrocardiography were monitored throughout the scan and ear protection was used, comprising earplugs molded from a silicone-based putty (President Putty, Coltene Whaledent, Mahwah, NJ, USA) placed in the external auditory meatus and neonatal earmuffs (MiniMuffs, Natus Medical Inc., San Carlos, CA, USA).

### 2.3 Image processing

HARDI data were pre-processed by manual removal of motion-corrupted volumes, PCA-based denoising (Veraart et al., 2016), susceptibility correction and eddy current and subject motion correction using FSL's topup-eddy algorithm (Andersson et al., 2016; Andersson et al., 2003; Andersson et al., 2017; Andersson and Sotiropoulos, 2016), bias field correction (Tustison et al., 2010) and intensity normalisation across datasets. ODF images were computed for each subject using multi-shell multi-tissue CSD. Tissue-specific group-average response functions for white matter and cerebrospinal fluid (CSF) were estimated using both b = 0 and b = 2500 s/mm2 shells. White matter fibre ODFs are assumed to be anisotropic and are modelled using a spherical harmonic series of order 8, whereas CSF is assumed to be isotropic and hence modelled using an ODF with spherical harmonic order 0. The white matter response function was estimated from single-fibre voxels identified using the algorithm described in (Tournier et al., 2013). To calculate the isotropic CSF response function, voxels within the ventricles were delineated by manually drawing regions of interest. The CSF and white matter response functions were averaged across subjects and used to calculate each subject's ODF images.

Structural MRI data were pre-processed by running bias field correction using the N4 algorithm (Tustison et al., 2010). T2-weighted images were brain extracted using BET from FSL (Smith, 2002) and segmented using an automated, neonatal-specific technique based on the DRAW-EM algorithm (Makropoulos et al., 2016). Tissue segmentations of the cortical grey matter, deep grey matter, white matter and ventricles were summed to calculate total brain volumes.

### 2.4 Fixel-based analysis

#### 2.4.1 Registration

Each infant's ODF image was registered to an iteratively refined, group-averaged ODF template using nonlinear transformations (Raffelt et al., 2012; Raffelt et al., 2011). The registration preserves the total ODF integral and the volume fractions of each fibre population (Raffelt et al., 2009). To avoid the effects of focal brain injury on the template morphology, the template was created from a subset of 36 subjects (20 male) without focal brain injury. The demographics of this subset are included in the Supplementary Table 1. A two-sample Kolgomorov-Smirnoff test was applied to test for differences between the template and the whole study group. No significant differences were found in any of the recorded perinatal characteristics (all p-values>0.05).

#### 2.4.2 Apparent fibre density

At high diffusion-weightings, the extra-axonal water is strongly attenuated and the total radial diffusion signal is proportional to the intra-axonal water. The amplitude of the ODF is proportional to the radial diffusion signal and therefore provides a measure of the intra-axonal volume fraction of the fibres aligned with the corresponding direction. FD was calculated for each fixel in each infant's warped ODF image. For each infant, the ODF lobes were segmented, and the FD of each lobe was calculated by numerically integrating the ODF lobe over the corresponding orientations (Smith et al., 2013). Fixels in each infant's warped ODF image were then reoriented to ensure orientation information remains anatomically consistent across voxels.

To achieve anatomical correspondence across subjects, each ODF in the template was segmented and a template fixel mask was defined within which to perform statistical analysis. The FD value from each infant's fixel was assigned to the corresponding fixel in the template.

#### 2.4.3 Fibre cross-section

At each point, the nonlinear mapping of each infant's ODF image to the template is given by the Jacobian matrix. The determinant of the Jacobian describes the local expansion or contraction with respect to the template. FC is a measure of the volume change in the direction perpendicular to the orientation of a fixel computed using the determinant of the Jacobian (Raffelt et al., 2017). FC is calculated using the warp from the template to the subject, and so a FC >1 represents a larger fibre bundle in the subject than in the template.

#### 2.4.4 Fibre density and cross-section

FD and FC can be combined to give a measure that reflects both changes in microscopic density and macroscopic morphology. For each fixel, FDC is calculated as FD multiplied by FC (Raffelt et al., 2017).

### 2.5 Statistical analysis

Whole-brain probabilistic tractography was performed in the ODF template, seeded from a whole-brain white matter mask to produce a tractogram of 100 million streamlines. From this tractogram, a subset of 10 million streamlines was selected that best fit the diffusion signal using the SIFT algorithm (Smith et al., 2013). Connectivity-based fixel enhancement (CFE) was performed. CFE uses probabilistic tractography to estimate the degree of structural connectivity between fixels, which is then used to smooth fixel measures across connected fixels and for multiple comparisons correction by identifying connected clusters of fixels of significant effect, similar to that of threshold-free cluster enhancement (Raffelt et al., 2015). Multiple comparisons correction was carried out using non-parametric permutation testing (Nichols and Holmes, 2002) with family-wise error (FWE) rate correction using the fixelcfestats command provided as part of MRtrix3. Following statistical analysis, an expert in neonatal neuroimaging (SJC) identified which tracts corresponded to those fixels that were significantly correlated to perinatal risk factors using FBA.

First, in order to assess the relationship between fixel-based measures and age at scan, regression analysis was performed between FD, FC and FDC and PMA. We then undertook regression analyses to assess the relationship between FD, FC and FDC and perinatal risk factors: GA at birth, the number of days on mechanical ventilation, the number of days on total parenteral nutrition (TPN) and birthweight z-scores. We also assessed group differences between male and female subjects. We carried out these analyses controlling for PMA at scan and GA at birth (except when GA at birth was the variable of interest and only PMA was included as a covariate). Morphological and microstructural differences have been observed previously between male and female subjects (Barnett et al., 2018; Gilmore et al., 2007) therefore we repeated this analysis including sex as an additional covariate.

FC and FDC may be associated with brain volume, and so we repeated our analyses with brain volume as a covariate. However, brain volume is also likely to be related to PMA at scan and all of the risk factors assessed here. We therefore assessed the relationship between brain volume and PMA, GA, days on ventilation, days on TPN, birthweight z-scores and sex differences using Pearson's correlation and report our findings in Supplementary Data (Supplementary Figs. 1–5 and Supplementary Table 2).

We did not assess the relationship between fixel-based measures and necrotising enterocolitis, chorioamnionitis or treatment for patent ductus arteriosus as there were few subjects in the study group (Table 1).

**Table 1:** Perinatal characteristics of the study group.

Perinatal clinical characteristic	All subjects	Male subjects n = 28	Female subjects n = 22	p-Value
Median (range) gestational age at birth (weeks)	30.4 (24.0–32.9)	29.9 (24.3–32.7)	30.0 (24.0–32.9)	0.3880
Median (range) postmenstrual age at scan (weeks)	42.1 (38.6–47.1)	42.1 (38.6–46.1)	41.6 (39.4–47.1)	0.8866
Median (range) birthweight (grams)	1202.5 (645–1990)	1215 (645–1990)	1130 (669–1960)	0.8044
Mean (SD) birthweight z-scores	−0.71 (0.876)	−0.72 (1.0)	−0.69 (0.70	0.9473
Median (range) days of ventilation	0 (0–40)	0 (0−30)	0 (0–40)	1
Median (range) days of total parenteral nutrition	6.5 (0–89)	6 (0–24)	6 (0–89)	0.9371
Rate of weight gain (grams per week)	164 (11–276)	166 (94–276)	146 (11–229)	0.5694
NEC requiring surgery (no, %)	1 (2%)	0 (0%)	1 (4.5%)	–
Chorioamnionitis (no., %)	2 (4%)	1 (3.6%)	1 (4.5%)	–
PDA requiring medical or surgical treatment (no., %)	1 (2%)	1 (3.6%)	0 (0%)	–

## 3 Results

### 3.1 Perinatal characteristics

The perinatal characteristics of the infants are described in Table 1.

### 3.2 MRI findings

Focal brain injury was observed on conventional MRI in three infants. One subject had a unilateral cerebellar haemorrhage; one subject had a small unilateral haemorrhagic parenchymal infarct, and one subject had haemorrhage in the right temporal lobe, cerebellar haemorrhage, cerebellar hypotrophy and bilateral intraventricular haemorrhage.

### 3.3 Relationship between fixel measures and postmenstrual age at scan

**Fig. 1:**
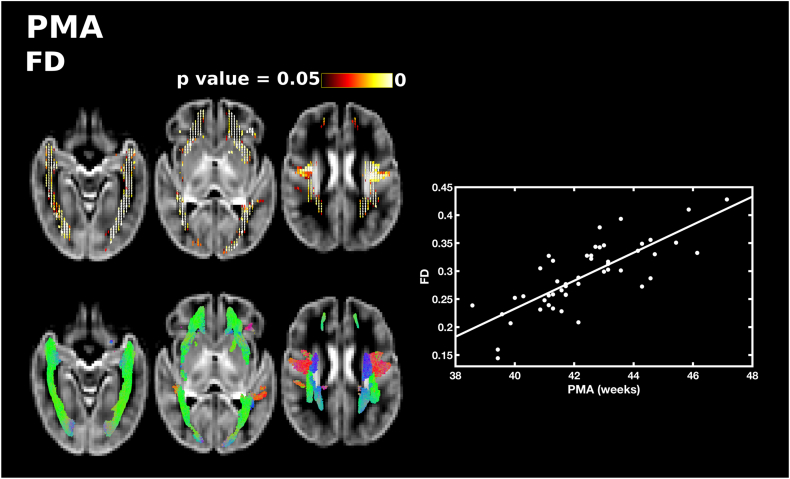
The relationship between PMA at scan and apparent fibre density (FD). Fixels with a significant positive correlation (corrected p < 0.05) are shown on the top row, and streamlines passing through significant fixels (coloured by direction red: left-right; green: anterior-posterior; blue: inferior-superior) are shown on the bottom row, in the axial plane. The scatter plot shows the correlation between PMA and FD averaged over all significant fixels. (For interpretation of the references to color in this figure legend, the reader is referred to the web version of this article.)

**Fig. 2:**
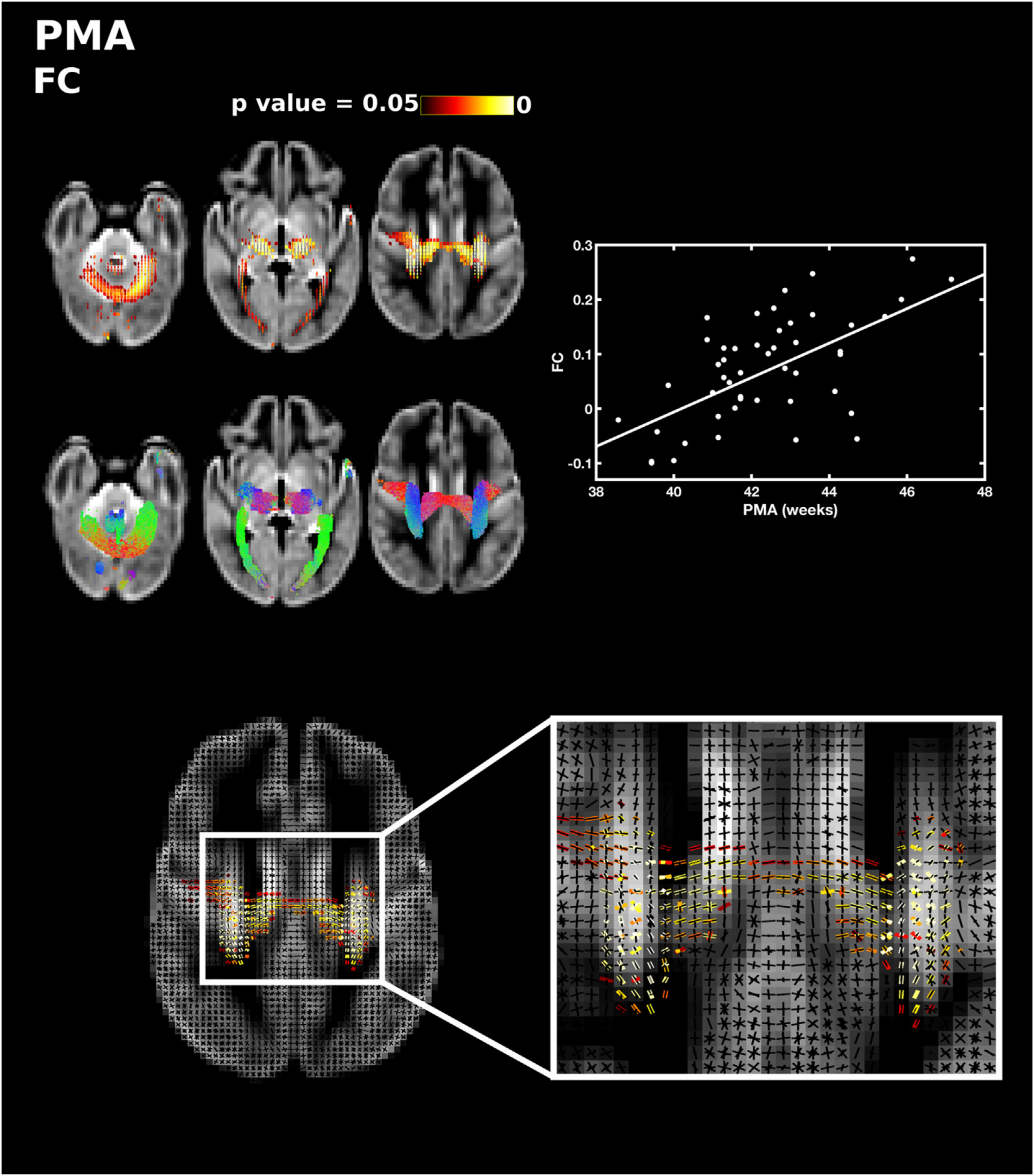
The relationship between PMA at scan and fibre cross-section (FC). Fixels with a significant positive correlation (corrected p < 0.05) are shown on the top row, and streamlines passing through significant fixels (coloured by direction red: left-right; green: anterior-posterior; blue: inferior-superior) are shown on the bottom row, in the axial plane. The scatter plot shows the correlation between PMA and FC averaged over all significant fixels. The single slice axial plane shows a close up of fixels within a crossing fibre region that are significantly correlated with PMA (red-yellow) overlaid on the fixel template (black), showing that callosal and corticospinal fixels are significantly associated with PMA but not association fibre fixels. (For interpretation of the references to color in this figure legend, the reader is referred to the web version of this article.)

**Fig. 3:**
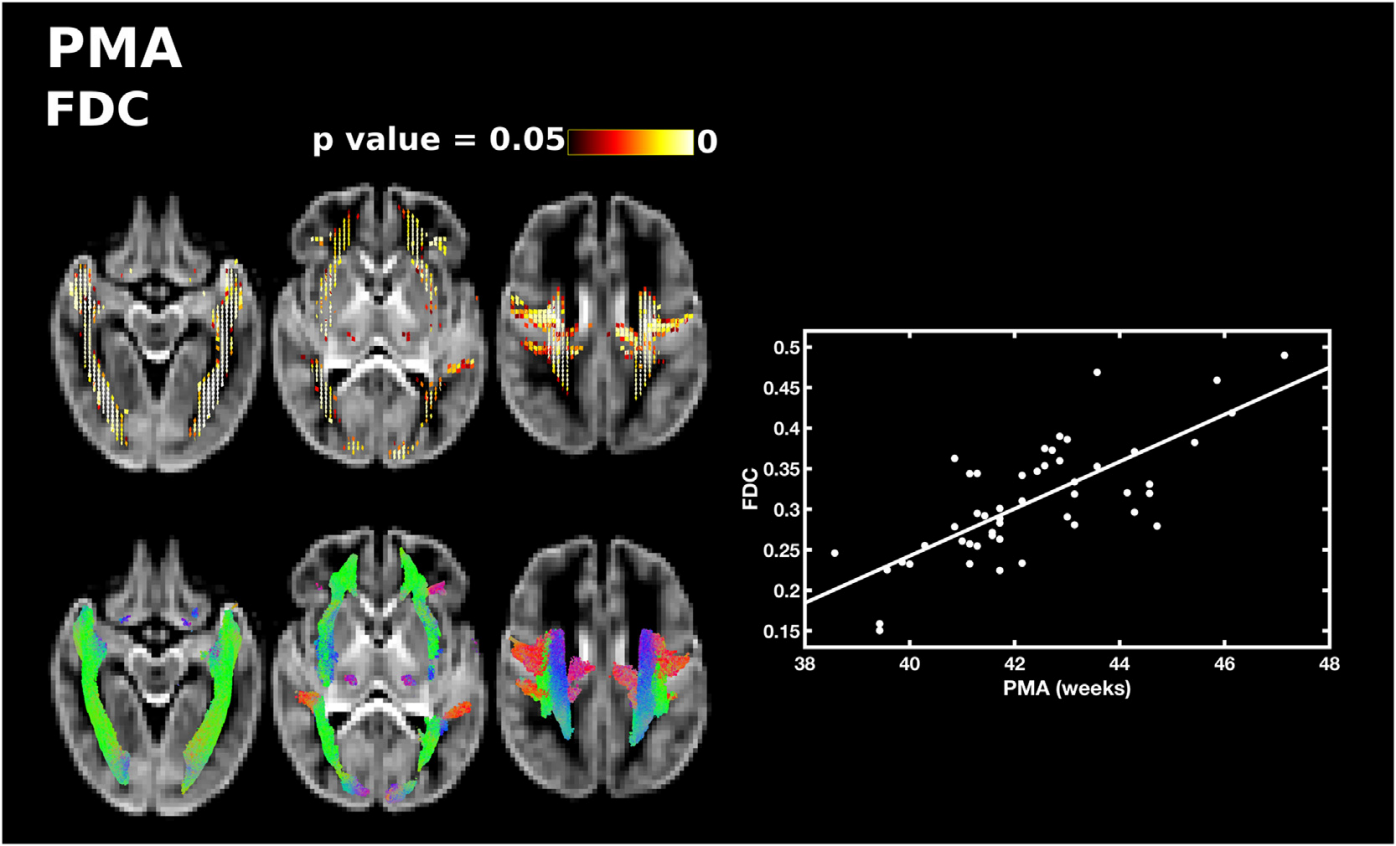
The relationship between PMA at scan and fibre density and cross-section (FDC). Fixels with a significant positive correlation (corrected p < 0.05) are shown on the top row, and streamlines passing through significant fixels (coloured by direction red: left-right; green: anterior-posterior; blue: inferior-superior) are shown on the bottom row, in the axial plane. The scatter plot shows the correlation between PMA and FDC averaged over all significant fixels. (For interpretation of the references to color in this figure legend, the reader is referred to the web version of this article.)

PMA at scan was significantly positively correlated with FD, FC and FDC throughout the white matter. PMA was correlated with FD in the forceps minor and forceps major, lateral projections of the corpus callosum, the corona radiata, inferior fronto-occipital fasciculus, superior longitudinal fasciculus and inferior longitudinal fasciculus (Fig. 1). There was a positive correlation between PMA and FC in the body of the corpus callosum, the corticospinal tract, inferior longitudinal fasciculus and cerebellum (Fig. 2). FDC was correlated with PMA in the middle portion of the body of the corpus callosum, the lateral projections of the corpus callosum, the forceps minor, forceps major, corticospinal tract, inferior fronto-occipital fasciculus, superior longitudinal fasciculus, inferior longitudinal fasciculus, thalamic regions and cerebellum (Fig. 3). There were no differences in the results when sex was added as a covariate.

### 3.4 Relationship between fixel measures and perinatal risk factors after controlling for postmenstrual age at scan and gestational age at birth

All the results reported below are bilateral, unless the hemisphere is stated, significant with FWE-corrected p-values <.05 and corrected for PMA at scan and GA at birth. The results are summarised in Table 2. There were no differences in the results when sex was included as a covariate.

**Table 2:** Summary of results from fixel-based analysis. All significant results are bilateral, unless the hemisphere is stated explicitly.

Variable of interest	FD	FC	FDC
PMA (positive correlation)	Forceps minor	Body of corpus callosum	Body of corpus callosum
	Forceps major	Corticospinal tract	Lateral projections of corpus callosum
	Lateral projections of the corpus callosum	Inferior longitudinal fasciculus	Forceps minor
	Corona radiata	Cerebellum	Forceps major
	Inferior fronto-occipital fasciculus		Corticospinal tract
	Superior longitudinal fasciculus		Inferior fronto-occipital fasciculus
	Inferior longitudinal fasciculus		Superior longitudinal fasciculus
			Inferior longitudinal fasciculus
			Thalamic regions
			Cerebellum
GA at birth (corrected for PMA; positive correlation)	splenium of corpus callosum	Genu of corpus callosum	Genu of corpus callosum
	tapetum of corpus callosum	Splenium of corpus callosum	Splenium of corpus callosum
	Left inferior fronto-occipital fasciculus	Anterior commissure	Tapetum of corpus callosum
		Right corticospinal tract	Anterior commissure
		Inferior fronto-occipital fasciculus	Inferior fronto-occipital fasciculus
		Superior longitudinal fasciculus	Inferior longitudinal fasciculus
		Inferior longitudinal fasciculus	Fornix
		Left Cingulum	
		Fornix	
		Thalamic regions	
		Cerebellum	
		Pons	
Days on ventilation (corrected for PMA and GA; negative correlation)	Cerebellum	Genu of corpus callosum	Corticospinal tract
	Pons	Splenium of corpus callosum	Inferior longitudinal fasciculus
		Tapetum of corpus callosum	Right Fornix
		Anterior commissure	Cerebellum
		Corticospinal tract	Pons
		Anterior limb of the internal capsule	
		Inferior fronto-occipital fasciculus	
		Inferior longitudinal fasciculus	
		Fornix	
		Thalamic regions	
		Cerebellum	
		Pons	
Days on total parenteral nutrition (corrected for PMA and GA; negative correlation)	NS	Corticospinal tract	NS
		External capsule	
		Cerebellum	
		Pons	
Birth weight z score (corrected for PMA and GA; negative correlation)	NS	Splenium of corpus callosum	Splenium of corpus callosum
		Corticospinal tract	Corticospinal tract
		Superior longitudinal fasciculus	Inferior fronto-occipital fasciculus
		Inferior longitudinal fasciculus	Inferior longitudinal fasciculus
		Cingulum	Right Fornix
		Fornix	
		Cerebellum	
		Pons	
Sex differences (male > female)	NS	Splenium of corpus callosum	Corticospinal tract
		Corticospinal tract	
		Inferior fronto-occipital fasciculus	
		Superior longitudinal fasciculus	
		Cingulum	
		Fornix	
		Inferior longitudinal fasciculus	
		Cerebellum	
Sex differences (female > male)	NS	NS	NS

NS = not significant.

#### 3.4.1 Gestational age at birth

**Fig. 4:**
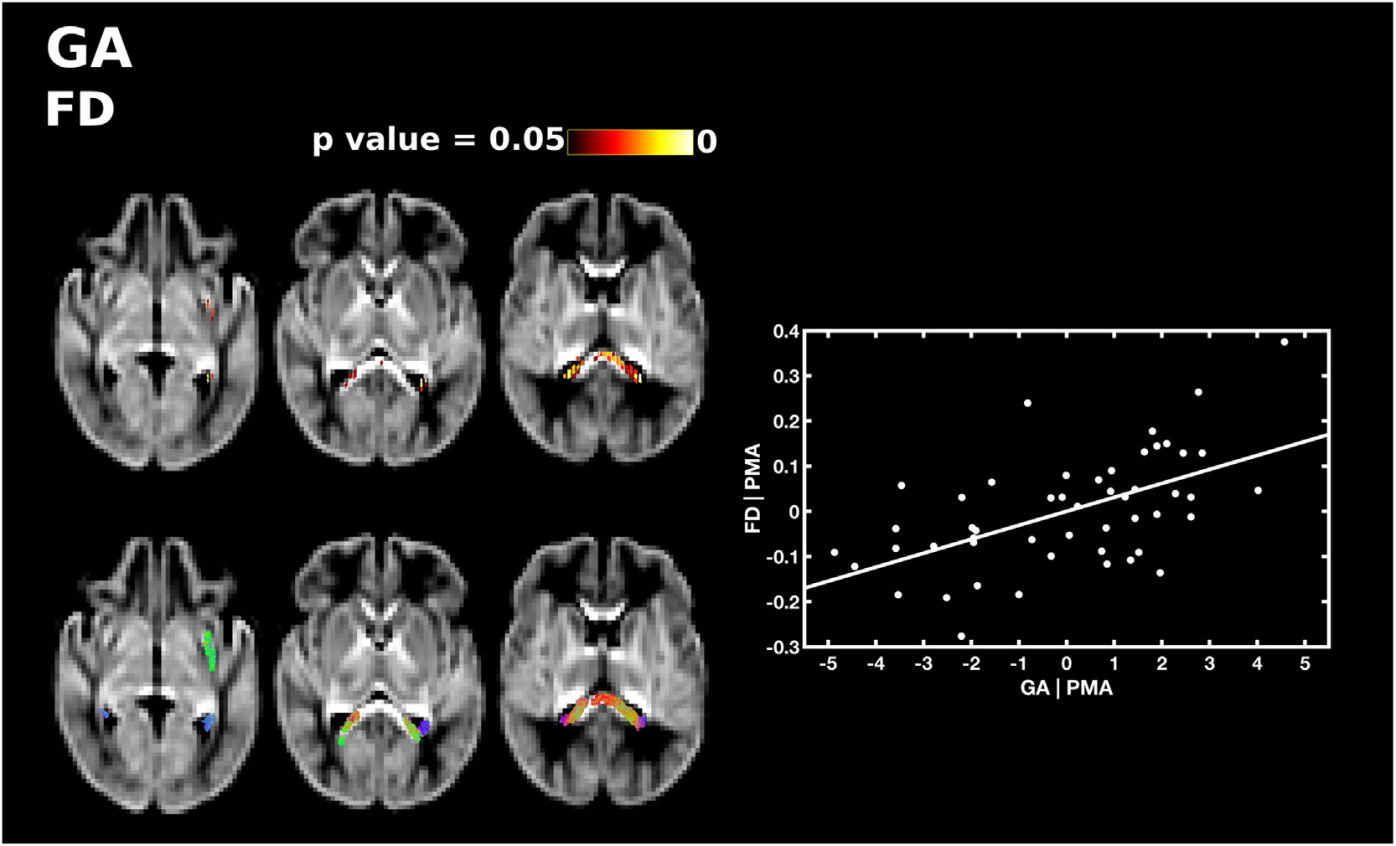
The relationship between GA at birth and apparent fibre density (FD), corrected for PMA at scan. Fixels with a significant positive correlation (corrected p < 0.05) are shown on the top row, and streamlines passing through significant fixels (coloured by direction red: left-right; green: anterior-posterior; blue: inferior-superior) are shown on the bottom row, in the axial plane. The scatter plot shows the partial correlation between GA and FD averaged over all significant fixels, corrected for PMA. (For interpretation of the references to color in this figure legend, the reader is referred to the web version of this article.)

**Fig. 5:**
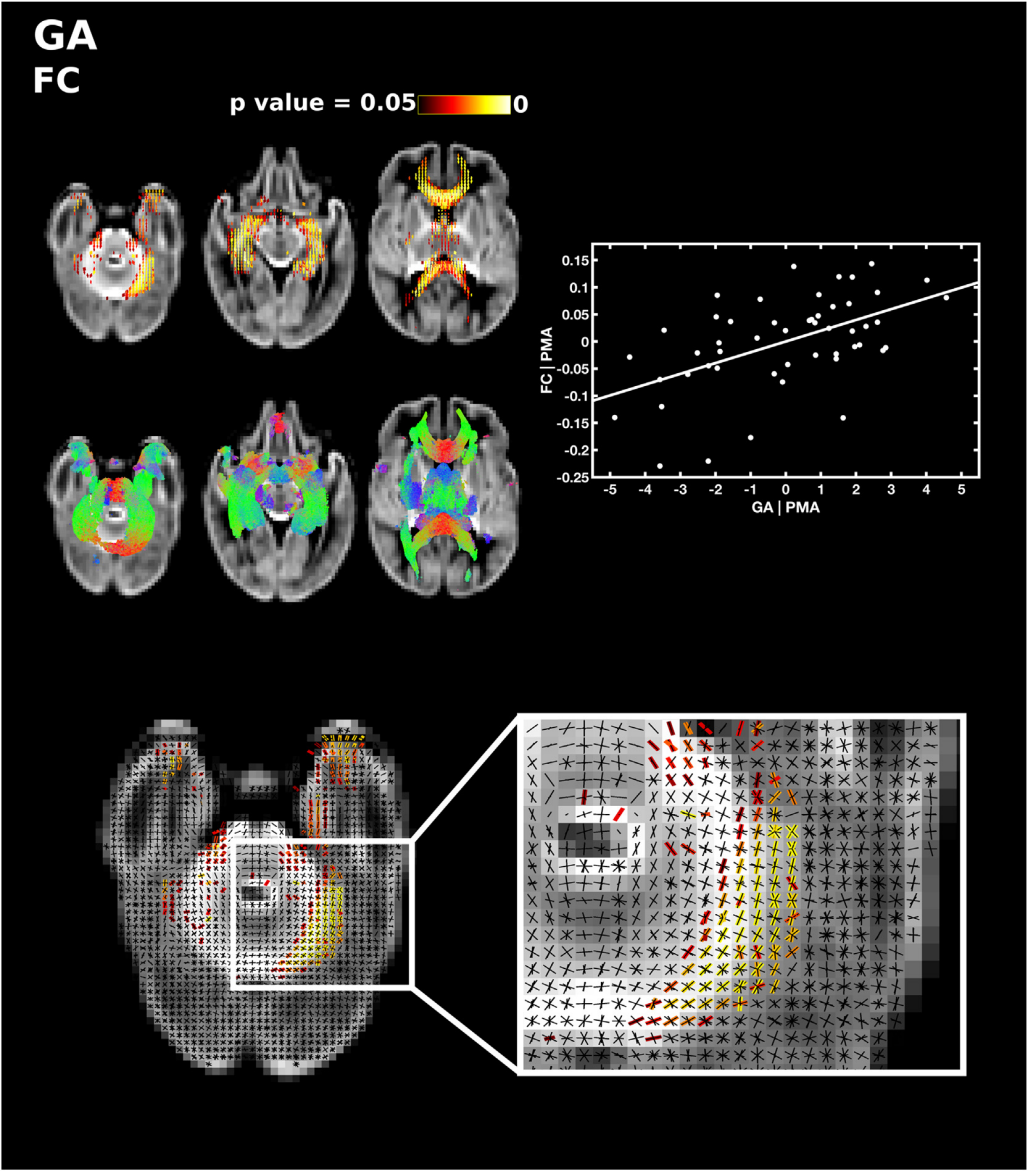
The relationship between GA at birth and fibre cross-section (FC), corrected for PMA at scan. Fixels with a significant positive correlation (corrected p < 0.05) are shown on the top row, and streamlines passing through significant fixels (coloured by direction red: left-right; green: anterior-posterior; blue: inferior-superior) are shown on the bottom row, in the axial plane. The scatter plot shows the partial correlation between GA and FC averaged over all significant fixels, corrected for PMA. The single slice axial plane in the FC panel shows a close up of fixels within the cerebellum that are significantly correlated with GA (red-yellow) overlaid on the fixel template (black), highlighting which fibres within crossing fibre regions are significantly associated with prematurity. (For interpretation of the references to color in this figure legend, the reader is referred to the web version of this article.)

**Fig. 6:**
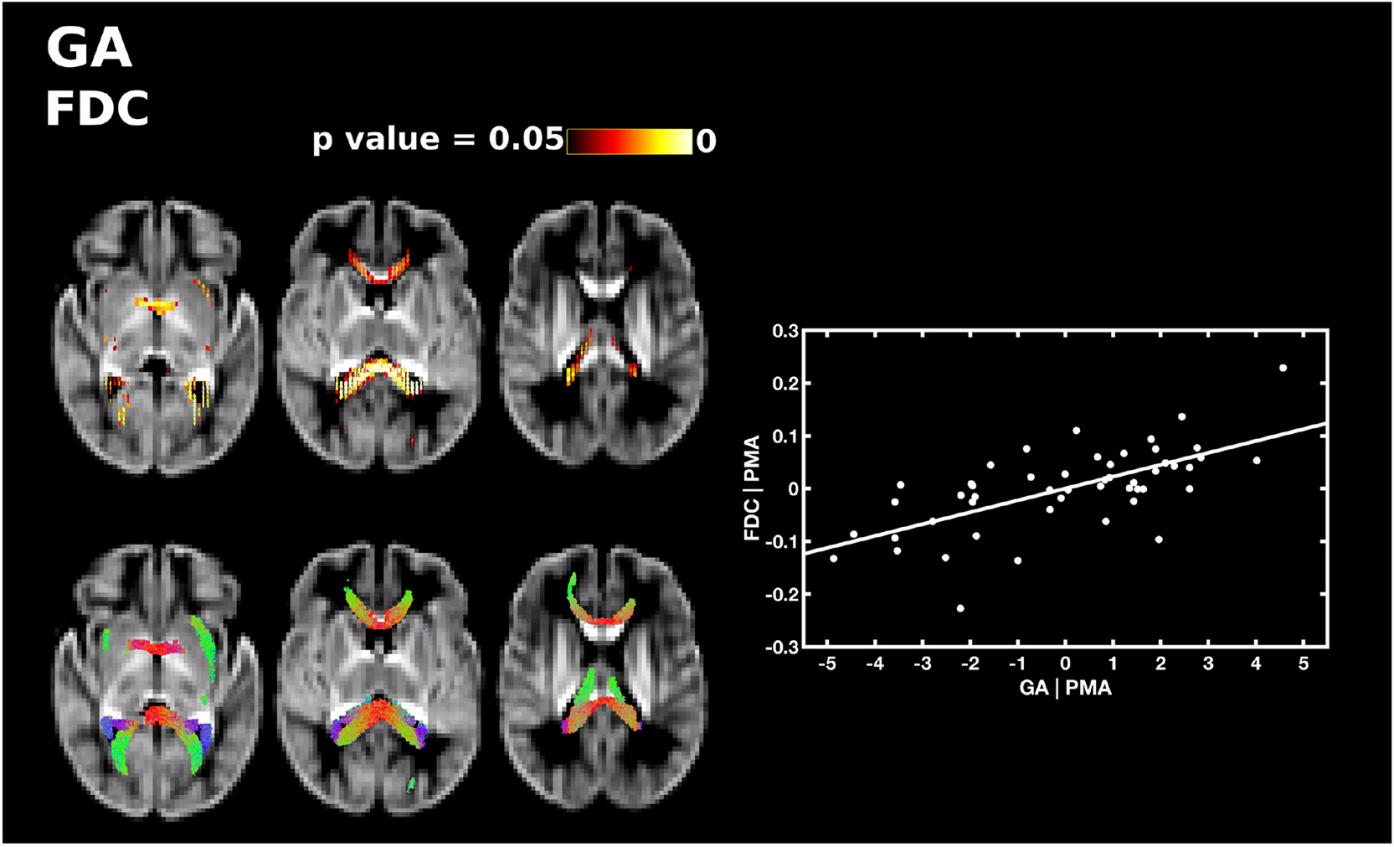
The relationship between GA at birth and apparent fibre density and cross-section (FDC), corrected for PMA at scan. Fixels with a significant positive correlation (corrected p < 0.05) are shown on the top row, and streamlines passing through significant fixels (coloured by direction red: left-right; green: anterior-posterior; blue: inferior-superior) are shown on the bottom row, in the axial plane. The scatter plot shows the partial correlation between GA and FDC averaged over all significant fixels, corrected for PMA. (For interpretation of the references to color in this figure legend, the reader is referred to the web version of this article.)

GA at birth was significantly positively correlated with FD, FC and FDC, after correcting for PMA at scan. GA was positively correlated with FD in the splenium 15 and tapetum of the corpus callosum, and a small region in the anterior portion of the left inferior fronto-occipital fasciculus (Fig. 4). GA was positively correlated with FC in the genu and splenium of the corpus callosum, the anterior commissure, the right corticospinal tract, the inferior fronto-occipital fasciculus, superior longitudinal fasciculus, inferior longitudinal fasciculus, fornix, cerebellum and pons (Fig. 5). FDC was positively correlated with GA in the genu, splenium and tapetum of the corpus callosum, the anterior commissure, and the middle-to-anterior regions of the inferior fronto-occipital fasciculus and inferior longitudinal fasciculus, and fornix (Fig. 6).

#### 3.4.2 Days on mechanical ventilation

**Fig. 7:**
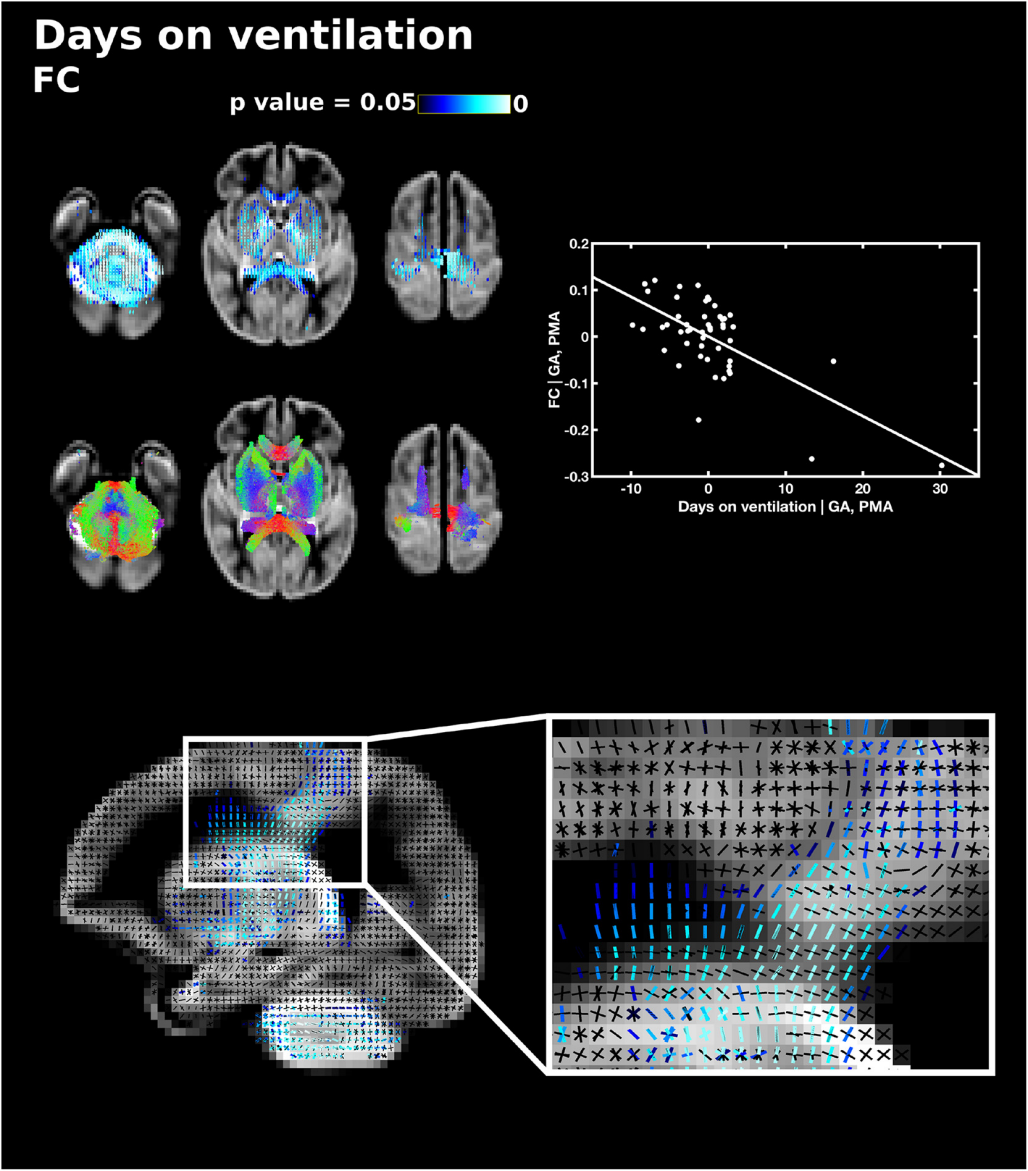
The relationship between the number of days requiring mechanical ventilation and fibre cross-section (FC), corrected for PMA at scan and GA at birth. Fixels with a significant negative correlation (corrected p < 0.05) are shown on the top row, and streamlines passing through significant fixels (coloured by direction red: left-right; green: anterior-posterior; blue: inferior-superior) are shown on the bottom row, in the axial plane. The scatter plot shows the partial correlation between days on mechanical ventilation and FC averaged over all significant fixels, corrected for PMA and GA. The sagittal plane shows a close up of fixels significantly correlated with days on ventilation (blue) overlaid on the fixel template (black) in the centrum semiovale, showing that only corticospinal fixels and not callosal or association fibre fixels are associated with days on ventilation. (For interpretation of the references to color in this figure legend, the reader is referred to the web version of this article.)

All fixel measures were significantly negatively correlated with the number of days requiring mechanical ventilation, corrected for PMA and GA. FD in the cerebellum and the pons was negatively correlated with number of days on mechanical ventilation (Supplementary Fig. 1). FC was negatively correlated with number of days on ventilation in the genu, splenium and tapetum of the corpus callosum, the anterior commissure, corticospinal tract, anterior limb of the internal capsule, middle-to-anterior regions of the inferior fronto-occipital fasciculus and inferior longitudinal fasciculus, fornix, thalamic regions, cerebellum and pons (Fig. 7). Of note, FBA was able to delineate which fibre bundles within a region of crossing fibres were correlated with mechanical ventilation, and highlighted that within the centrum semiovale, duration of mechanical ventilation was significantly negatively correlated with FC in fibres from the corticospinal tract and not association fibres or lateral projections of the corpus callosum. FDC was negatively correlated with number of days on mechanical ventilation in the corticospinal tract, inferior longitudinal fasciculus, the right fornix, the cerebellum and pons (Supplementary Fig. 2).

#### 3.4.3 Days on total parenteral nutrition

**Fig. 8:**
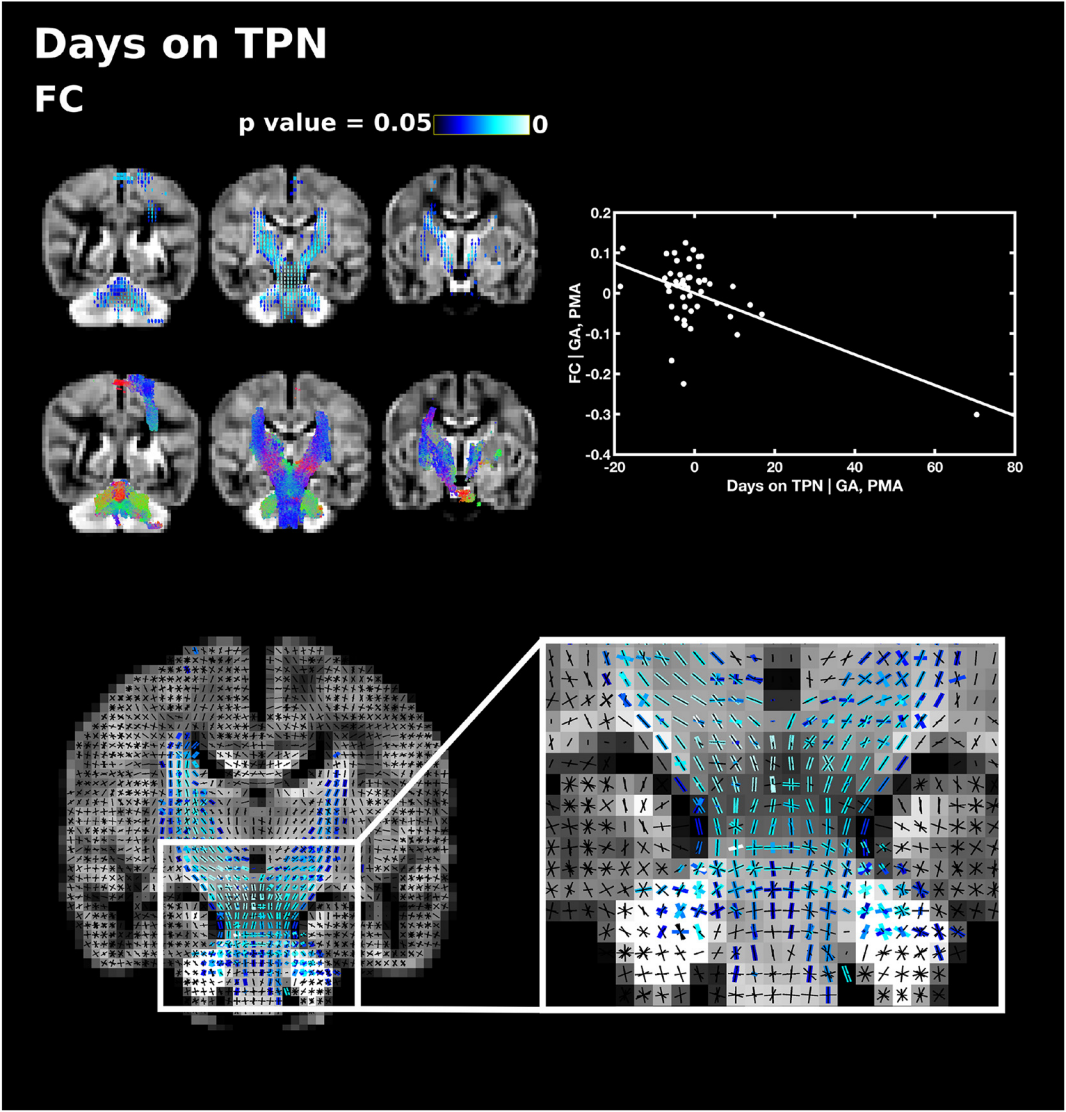
The relationship between the number of days on total parenteral nutrition (TPN) and fibre cross-section (FC), corrected for PMA at scan and GA at birth. Fixels with a significant negative correlation (corrected p < .05) are shown on the top row, and streamlines passing through significant fixels (coloured by direction red: left-right; green: anterior-posterior; blue: inferior-superior) are shown on the bottom row, in the coronal plane. The scatter plot shows the partial correlation between days TPN and FC averaged over all significant fixels, corrected for PMA and GA. The single slice coronal plane shows a close up of fixels significantly correlated with days on TPN (blue) overlaid on the fixel template (black) in the brainstem, showing that both corticospinal fibres and pontine fixels are associated with days on TPN. (For interpretation of the references to color in this figure legend, the reader is referred to the web version of this article.)

There were no significant correlations between FD, FDC and the number of days requiring TPN. FC showed a negative correlation in the corticospinal tract, external capsule, cerebellum and pons (Fig. 8). We performed post-hoc analysis to determine whether these results were influenced by an outlier. After removing the subject with highest number of days on TPN (89 days), the results were no longer statistically significant.

#### 3.4.4 Birthweight

**Fig. 9:**
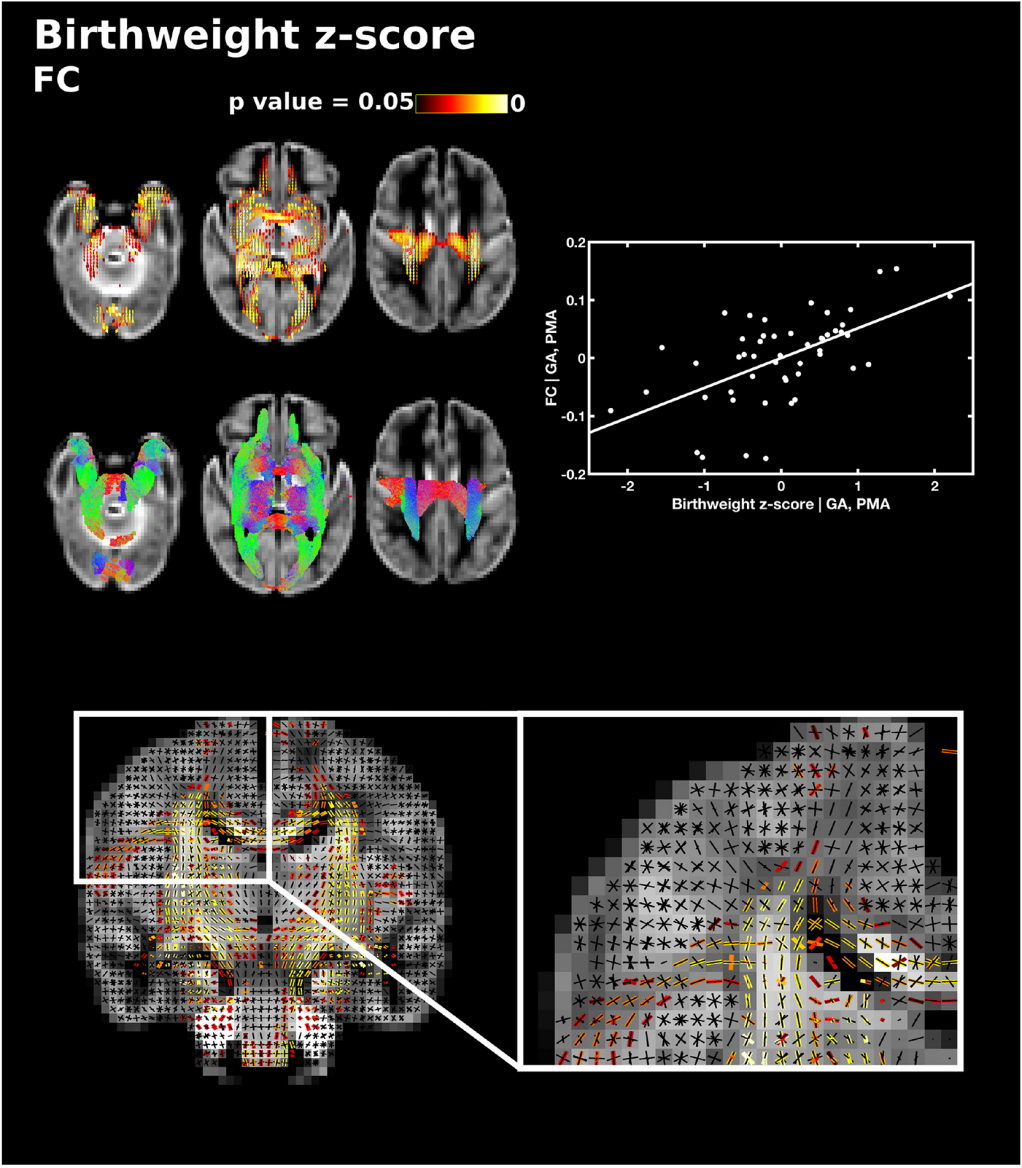
The relationship between birthweight z-scores and fibre cross-section (FC), corrected for PMA at scan and GA at birth. Fixels with a significant positive correlation (corrected p < 0.05) are shown on the top row, and streamlines passing through significant fixels (coloured by direction red: left-right; green: anterior-posterior; blue: inferior-superior) are shown on the bottom row, in the axial plane. The scatter plot shows the partial correlation between birthweight z-scores and FC averaged over all significant fixels, corrected for PMA and GA. The single slice coronal plane shows a close up of fixels significantly correlated with birthweight z-scores (red-yellow) overlaid on the fixel template (black) in the centrum semiovale, showing that corticospinal and callosal fixels are associated with birthweight z-scores, but not association fibre fixels. (For interpretation of the references to color in this figure legend, the reader is referred to the web version of this article.)

Birthweight z-scores were not correlated with FD. FC was significantly positively correlated with birthweight z-scores throughout the white matter including the genu, splenium, body and lateral projections of the corpus callosum, the anterior commissure, corticospinal tract, the right anterior limb of the internal capsule, inferior fronto-occipital fasciculus, superior longitudinal fasciculus, inferior longitudinal fasciculus, fornix, thalamic regions, the cerebellum and pons (Fig. 9). FDC was positively correlated with birthweight z-scores in the splenium of the corpus callosum, corticospinal tract, inferior fronto-occipital fasciculus, inferior longitudinal fasciculus and the right fornix (Supplementary Fig. 3).

#### 3.4.5 Sex

**Fig. 10:**
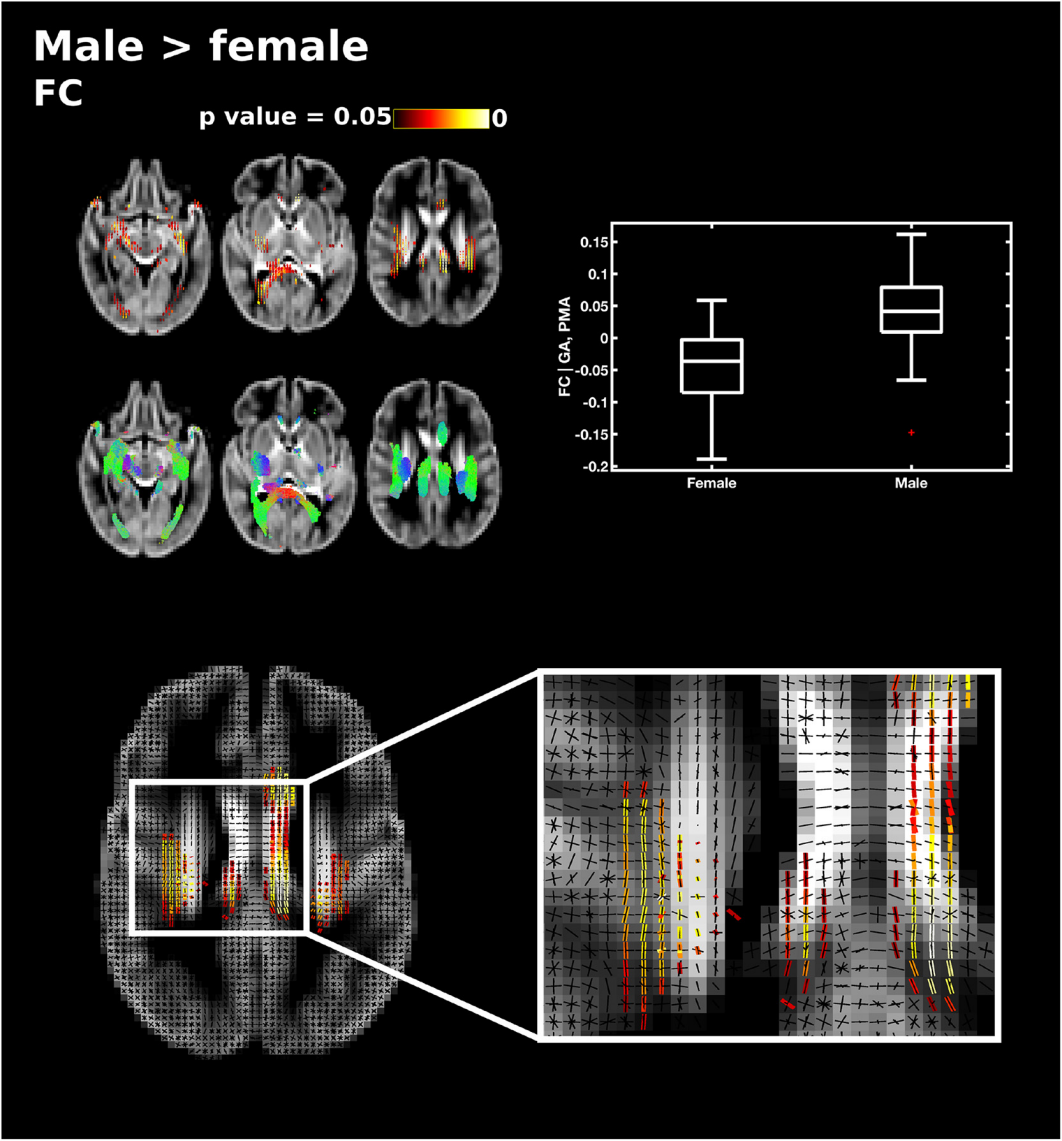
Differences in fibre cross-section (FC) between male and female subjects, corrected for PMA at scan and GA at birth. Fixels with significantly higher FC in male subjects (corrected p < 0.05) are shown on the top and streamlines passing through significant fixels (coloured by direction red: left-right; green: anterior-posterior; blue: inferior-superior) are shown on the bottom row in the axial plane. The boxplot shows FC values for male and female subjects averaged over all significant fixels, corrected for GA and PMA. The single slice axial plane shows a close up of significant fixels (red-yellow) overlaid on the fixel template (black), showing that fixels in cingulum and superior longitudinal fasciculus but not corpus callosum differ between male and female subjects. (For interpretation of the references to color in this figure legend, the reader is referred to the web version of this article.)

There were no significant differences in FD between male and female subjects. Male subjects showed significantly higher FC in the splenium of the corpus callosum, corticospinal tract, the superior longitudinal fasciculus, inferior longitudinal fasciculus, cingulum, fornix and cerebellum (Fig. 10) and higher FDC in the corticospinal tract at the level of the centrum semiovale and posterior limb of internal capsule (Supplementary Fig. 4). There were no instances where fixel measures were significantly higher in female subjects than male subjects.

## 4 Discussion

This study reveals fibre population-specific associations between measures of fibre density and regional white matter fasciculi cross-section, and perinatal risk factors. Our findings are in line with previous dMRI studies by our group and others assessing white matter development in the preterm population, demonstrating abnormal white matter development associated with immaturity at birth, longer requirement for mechanical ventilation, parenteral nutrition, stress and illness severity (Anjari et al., 2009; Ball et al., 2010; Barnett et al., 2018; Brummelte et al., 2012; Chau et al., 2009; Zwicker et al., 2013). Using FBA, we demonstrate that microstructural alterations in fibre density and macrostructural alterations in fibre bundle cross-section associated with clinical risk factors affect individual fibre bundles rather than spatially contiguous voxels. In the unmyelinated neonatal brain, lower FC reflects a reduction in the cross-sectional area of a white matter tract and lower FD indicates a reduction in the intra-axonal volume of fibres orientated in a particular direction, presumably due to axons with smaller diameter or fewer axons, although it is not possible to differentiate these two possibilities using this approach. Nevertheless, both reduced FD and FC are likely to reflected impaired white matter development and impaired ability to transfer information across brain regions.

In general, the correlations between perinatal risk factors and FC were more widespread than the correlations with FD. Indeed, there were no correlations between FD and the number of days requiring TPN or birthweight z-scores, and no significant differences in FD were found between male and female subjects. These results suggest that the perinatal clinical variables studied here are more strongly associated with reductions in the cross-sectional area of white matter fasciculi rather than changes in apparent fibre density. It is possible that impaired white matter development previously attributed to microstructural changes in DTI studies may be due to alterations in fibre cross-sectional area of specific fibre bundles at the macroscopic scale. This study extends our knowledge of morphological changes associated with perinatal risk factors by demonstrating fibre-specific relationships. Using fixel-based measures it is possible to identify whether morphological changes are localised to specific fibre bundles or are related to changes across the whole of the white matter. This is of particular importance in regions of crossing fibres. For example, in the centrum semiovale, we observed that FC in the corticospinal tract was diminished with greater requirement for mechanical ventilation (when correcting for PMA and GA), however there was no relationship between duration of mechanical ventilation and FC in the association fibres traversing the region. By identifying which tracts, and therefore which functional pathways, are affected by specific risk factors it may be possible to anticipate future developmental impairment.

After including total brain volume, correlations between fixel measures and GA at birth, days on TPN, birthweight z-scores and differences between male and female subjects were no longer statistically significant. However, brain volume was strongly correlated with age at scan and the perinatal risk factors. Including brain volume as a covariate, therefore, essentially removes the effect of interest. Future FBA studies may extend to multivariate analyses, taking into account the interdependencies of variables. After including sex as a covariate in addition to PMA and GA there were no changes to the results and no significant sex differences were observed in any of the risk factors studied here. Therefore, it is unlikely that changes in fixel measures observed across risk factors are driven by sex-specific morphological differences.

Previous DTI studies have shown a dose-dependent relationship between GA at birth and white matter FA values (Anjari et al., 2007) and preterm birth is associated with reduced white matter volume (Inder et al., 2005; Kersbergen et al., 2016a; Srinivasan et al., 2006; Thompson et al., 2007). In a recent study Pannek et al. (2018) showed that GA at birth was positively correlated with all three fixel measures in the splenium of the corpus callosum. In our study, GA at birth was positively correlated with FD in the splenium and tapetum of the corpus callosum and the anterior left inferior fronto-occipital fasciculus and FDC was positively correlated with GA in the genu, splenium and tapetum of the corpus callosum, anterior commissure, inferior fronto-occipital fasciculus, inferior longitudinal fasciculus and fornix, when correcting for PMA at scan. These results suggest that commissural fibres, and association fibres to a lesser extent, are vulnerable to disturbances in intra-axonal volume fraction following preterm birth.

Respiratory illness during the perinatal period is associated with poor developmental outcome (Hansen et al., 2004; Short et al., 2003) and, independent of prematurity at birth, is associated with white matter abnormalities identified on DTI (Anjari et al., 2009; Ball et al., 2010) and reduced white matter and cerebellar volumes in preterm infants (Argyropoulou et al., 2003; Boardman et al., 2007; Thompson et al., 2007) and preterm-born children (Reiss et al., 2004). FBA reveals that, while the relationship between days on ventilation and FC was widespread across the white matter and cerebellum, microstructural changes in FD were localised to the cerebellum and pons, and alterations in FDC were observed in the corticospinal tract, cerebellum, pons and inferior longitudinal fasciculus. Cerebellar microstructural development in preterm infants has been not been well characterised. Brossard-Racine et al. (2017) reported counterintuitive higher FA and lower MD associated with compromised respiratory function in the dentate nucleus and white matter abnormality scores in the vermis. This is most likely due the prevalence of crossing fibres in these regions, which have been demonstrated in post-mortem foetal brains using CSD tractography (Takahashi et al., 2014), highlighting the need for higher-order diffusion models in regions of complex fibre configurations, and specifically the cerebellum. After correcting for brain volume, the association with FC remained statistically significant in the corticospinal tract, cerebellum and pons, and with FDC, in the pons. While it is of interest that respiratory illness was associated with alterations in white matter in respiratory neural networks including brain stem and cerebellum, the developing cerebellum is also vulnerable to hypoxic-ischemic injury (Sargent et al., 2004) and it is not possible in this study to determine causal relations.

Undernutrition is common in preterm infants and contributes to postnatal growth failure (Hay Jr., 2013; Su, 2014). Requirement for TPN can be an indicator of illness severity, as generally more critically ill infants require TPN for longer (Ehrenkranz et al., 2011). Barnett et al. (2018) found FA correlated negatively with duration of parenteral nutrition throughout the white matter in preterm infants imaged at TEA. Other studies have shown requirement of parenteral nutrition is associated with grey matter, white matter and cerebellar abnormalities assessed qualitatively on conventional MRI (Beauport et al., 2017; Brouwer et al., 2017; Kidokoro et al., 2013). FBA demonstrates a relative reduction in FC in the corticospinal tract, cerebellum and pons associated with longer duration of TPN, when correcting for PMA and GA. This overlaps with the regions of white matter associated with longer need for ventilation, suggesting these risk factors potentiate each other. This is supported by findings from Ball et al. (2017), showing that a multimodal imaging pattern which consisted of reduced cerebellar and brainstem volume, reductions in FA in the brain stem and corpus callosum and higher T2 signal intensity in the cerebellum was associated with markers of neonatal sickness, including days on ventilation and TPN.

Lower birth weight, specifically intrauterine growth restriction, is associated with reduced FA values in the corpus callosum (Padilla et al., 2014), reduced connectivity (Batalle et al., 2012) and with lower regional and whole brain volumes (Bruno et al., 2017; Kersbergen et al., 2016b). In this study, birthweight z-scores were positively correlated with FC and FDC across the white matter, but no microstructural changes in FD were observed. Similarly, Ball et al. (2017) found that a multivariate marker of intrauterine compromise, including intrauterine growth restriction, was associated with reductions in brain volume and higher T2 signal but with little contribution from microstructural measures.

We also explored differences in white matter microstructure and fasciculi cross-section between male and female preterm infants. Male sex has been implicated as a risk factor for poorer outcome in preterm infants (Hintz et al., 2006; Serenius et al., 2013; Wood et al., 2005). In this study, no microstructural differences were found between male and female subjects. The relationship between male sex and white matter alterations is not clear as some studies have shown no differences in diffusion measures between male and female preterm infants (Anjari et al., 2007; Skiold et al., 2014; van Kooij et al., 2012), other studies reported lower FA in in the splenium of the corpus callosum in preterm males (Rose et al., 2009), and we have observed higher diffusivity in male preterm infants compared to preterm females at TEA (Barnett et al., 2018). In this study, male infants had higher FC across the whole of the white matter, although this relationship was not observed when we corrected for brain volume (Skiold et al., 2014; Thompson et al., 2007).

FD, FC and FDC measures were positively correlated with age at scan throughout the white matter. These results are in agreement with the findings from Pannek et al. (2018), and a number of DTI studies showing increased FA (Ball et al., 2010; Pecheva et al., 2017; Rose et al., 2014) and volumetric studies showing increased white matter volume (Inder et al., 2005; Makropoulos et al., 2016) with increasing age during this period. We observed widespread maturational changes in FDC during the age range studied here. Such changes in FDC may better represent changes in the ability to relay information than measures of FD and FC alone (Raffelt et al., 2017).

While our findings describe local white matter damage, they are likely to also reflect grey matter damage leading to abnormal thalamo-cortical connections (Ball et al., 2013). Cortical maturation from 25 weeks PMA to term is marked by increased dendritic arborisation observable as greater geometric complexity on dMRI (Batalle et al., 2019; McKinstry et al., 2002). The development, or disruption, of cellular processes in the cortex is likely to affect white matter connections. Future studies assessing FBA and cortical development could contribute to improved understanding of the interplay between anomalous grey and white matter development observed in this population.

We recognise that this study is limited by a relatively small study group of preterm infants and larger studies with neurodevelopmental outcome measures in later childhood are required. Nevertheless, this study demonstrates that the FBA approach offers new insights into white matter development and injury related to perinatal risk factors.

## 5 Summary

In summary, this study shows that greater exposure to the perinatal risk factors studied here was associated with more wide-spread reductions in cross-section of white matter fasciculi than with alterations in fibre density, offering additional information to that which can be obtained from traditional diffusion measures of anisotropy and diffusivity. We demonstrate fibre bundle-specific relationships between risk factors and fixel measures, providing new insight into white matter development and injury in this vulnerable population.

## Appendix A Supplementary data

Supplementary material Image 1
