# Supplementary material for: Fixel-based analysis of the preterm brain: Disentangling bundle-specific white matter microstructural and macrostructural changes in relation to clinical risk factors
Source: Neuroimage Clin. 2019 Apr 10;23:101820. doi: 10.1016/j.nicl.2019.101820 (PMC6462822; doi:10.1016/j.nicl.2019.101820)
Supplement: Supplementary file 1 — Supplementary material [file mmc1.docx]

**Fixel based analysis of white matter fibre density and morphology in the preterm brain.**

Diliana Pecheva^1^, J-Donald Tournier^1^, Maximilian Pietsch^1^, Daan Christiaens^1^, Dafnis Batalle^1^, Daniel C. Alexander^2^, Joseph V Hajnal^1^, A. David Edwards^1^, Hui Zhang^2^, Serena J Counsell^1^.

^1^Centre for the Developing Brain, School of Biomedical Engineering & Imaging Sciences, King’s College London, UK

^2^Department of Computer Science and Centre for Medical Image Computing, University College London, UK

# Supplementary Methods

The characteristics of the subset of subjects included in the creation of the template are summarised in Supplementary Table 1.

Supplementary Table 1. Perinatal characteristics of the subset of subjects used to create the template.

| Perinatal clinical characteristic | All subjects | | Template subjects | p-value |
| --- | --- | --- | --- | --- |
| Median (range) gestational age at birth (weeks) | 30.4 (24.0-32.9) | | 30.4 (24-32.9) | 0.9995 |
| Median (range) postmenstrual age at scan (weeks) | 42.1 (38.6-47.1) | | 41.7 (39.4-45.9) | 0.8955 |
| Median (range) birthweight (grams) | 1202.5 (645-1990) | | 1217 (669-1990) | 0.9808 |
| Mean (SD) birthweight z-scores | -0.71 (0.876) | | -0.58 (0.94) | 0.9650 |
| Median (range) days of ventilation | 0 (0-40) | | 0 (0-40) | 0.9963 |
| Median (range) days of total parenteral nutrition | 6.5 (0-89) | | 7 (0-89) | 0.9995 |
| Rate of weight gain (grams per week) | 164 (11-276) | | 164 (11-267) | 1 |
| NEC requiring surgery (no, %) | 1 (2%) | | 1 (2.8%) | - |
| Chorioamnionitis (no., %) | 2 (4%) | | 0 (0%) | - |
| PDA requiring medical or surgical treatment (no., %) | 1 (2%) | 0 (0%) | |  |

# Supplementary Results

## Relationship between fixel measures brain volume

Brain volume was significantly positively correlated with FD, FC and FDC (Supplementary Figures 5-7), after correcting for PMA at scan. Brain volume and FD were correlated in the splenium of the CC, the anterior commissure, the left CST, the left ILF and the right fornix. Brain volume was significantly positively correlated with FC and FDC throughout the whole white matter.

## Relationship between fixel measures and perinatal risk factors after correcting for PMA at scan, GA at birth and total brain volume

After including brain volume as a covariate, the correlations between fixel measures and GA at birth, days on TPN, birthweight z-scores and differences between male and female subjects were no longer statistically significant.

### Days on mechanical ventilation

When brain volume was included as a covariate in addition to PMA and GA, the relationship between days on ventilation and FD was no longer statistically significant. FC was negatively correlated with number of days on mechanical ventilation in the CST (below the level of the PLIC), cerebellum and pons (Supplementary Figure 8) and FDC was negatively correlated with days requiring mechanical ventilation in the pons (Supplementary Figure 9).

## Correlations between brain volume and perinatal risk factors

Brain volume was significantly positively correlated with PMA at scan (𝜌=0.343, p-value=0.015). Brain volume was positively correlated with GA at birth but not statistically significant. (𝜌=0.187, p-value=0.195). Brain volume was significantly negatively correlated with days on ventilation (𝜌 =-0.408, p-value=0.003) and days on TPN (𝜌 =-0.411, p-value=0.003). Brain volume was positively correlated with birthweight z-scores and almost reached statistical significance (𝜌=0.274, p-value=0.054). Brain volume was greater in male subjects compared with female subjects (Wilcoxon rank sum test, p-value=0.015).

| 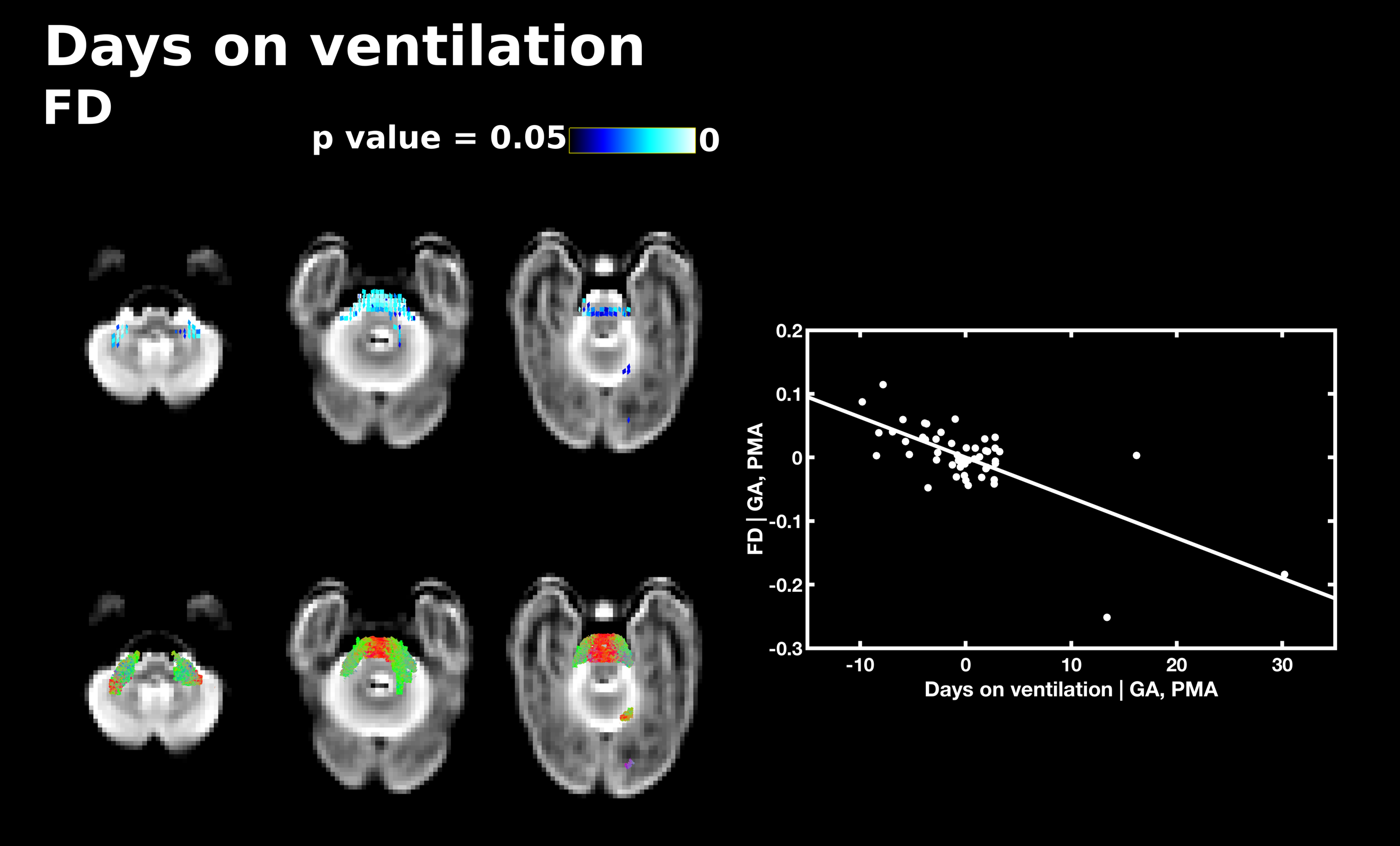  Supplementary Figure 1. The relationship between the number of days requiring mechanical ventilation and apparent fibre density (FD), corrected for PMA at scan and GA at birth. Fixels with a significant negative correlation (corrected p < 0.05) are shown on the top row, and streamlines passing through significant fixels (coloured by direction red: left-right; green: anterior-posterior; blue: inferior-superior) are shown on the bottom row, in the axial plane. The scatter plot shows the partial correlation between days on mechanical ventilation and FD averaged over all significant fixels, corrected for PMA and GA. |
| --- |

| 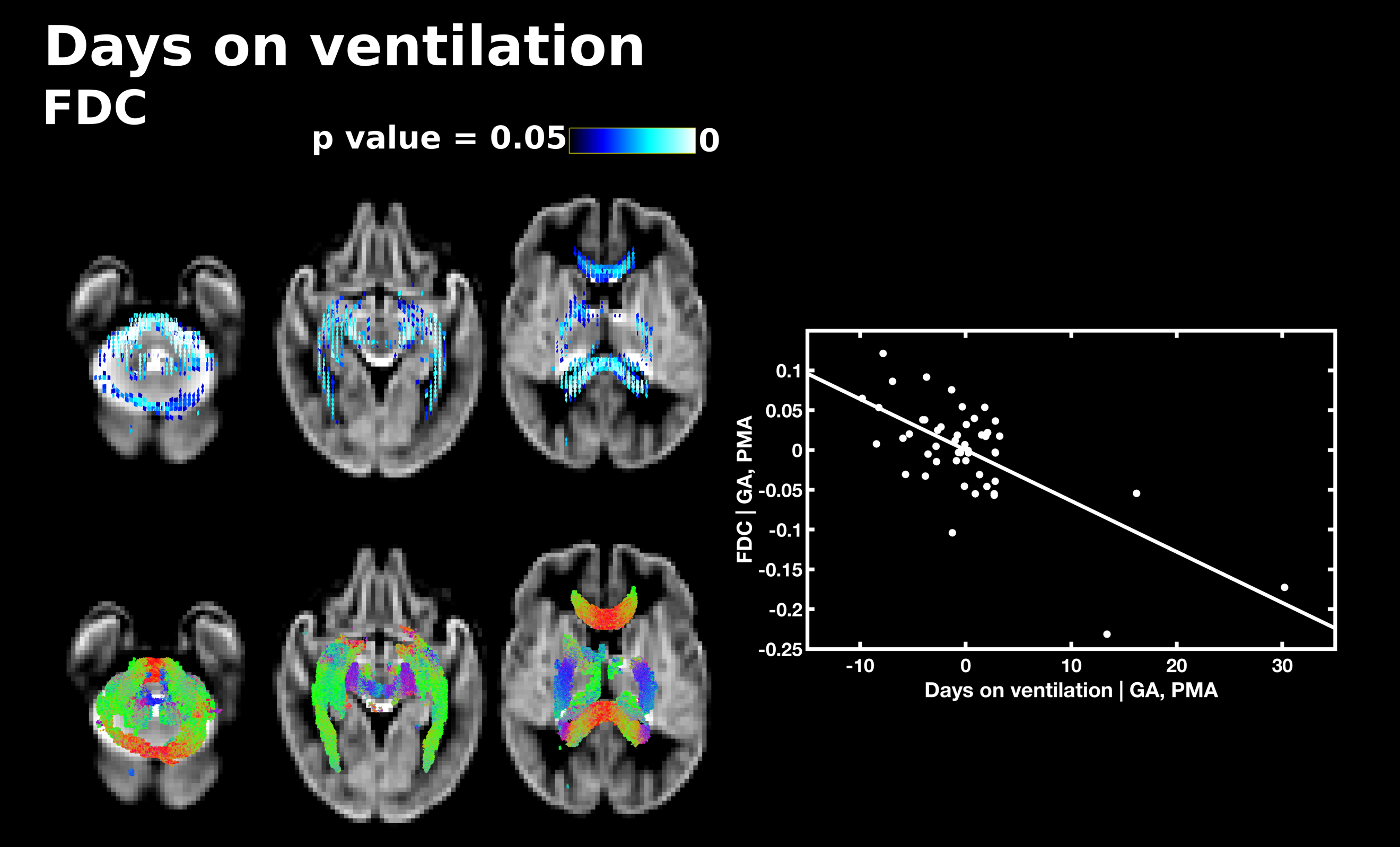  Supplementary Figure 2. The relationship between the number of days requiring mechanical ventilation and apparent fibre density and cross-section (FDC), corrected for PMA at scan and GA at birth. Fixels with a significant negative correlation (corrected p < 0.05) are shown on the top row, and streamlines passing through significant fixels (coloured by direction red: left-right; green: anterior-posterior; blue: inferior-superior) are shown on the bottom row, in the axial plane. The scatter plot shows the partial correlation between days on mechanical ventilation and FDC averaged over all significant fixels, corrected for PMA and GA. |
| --- |

| 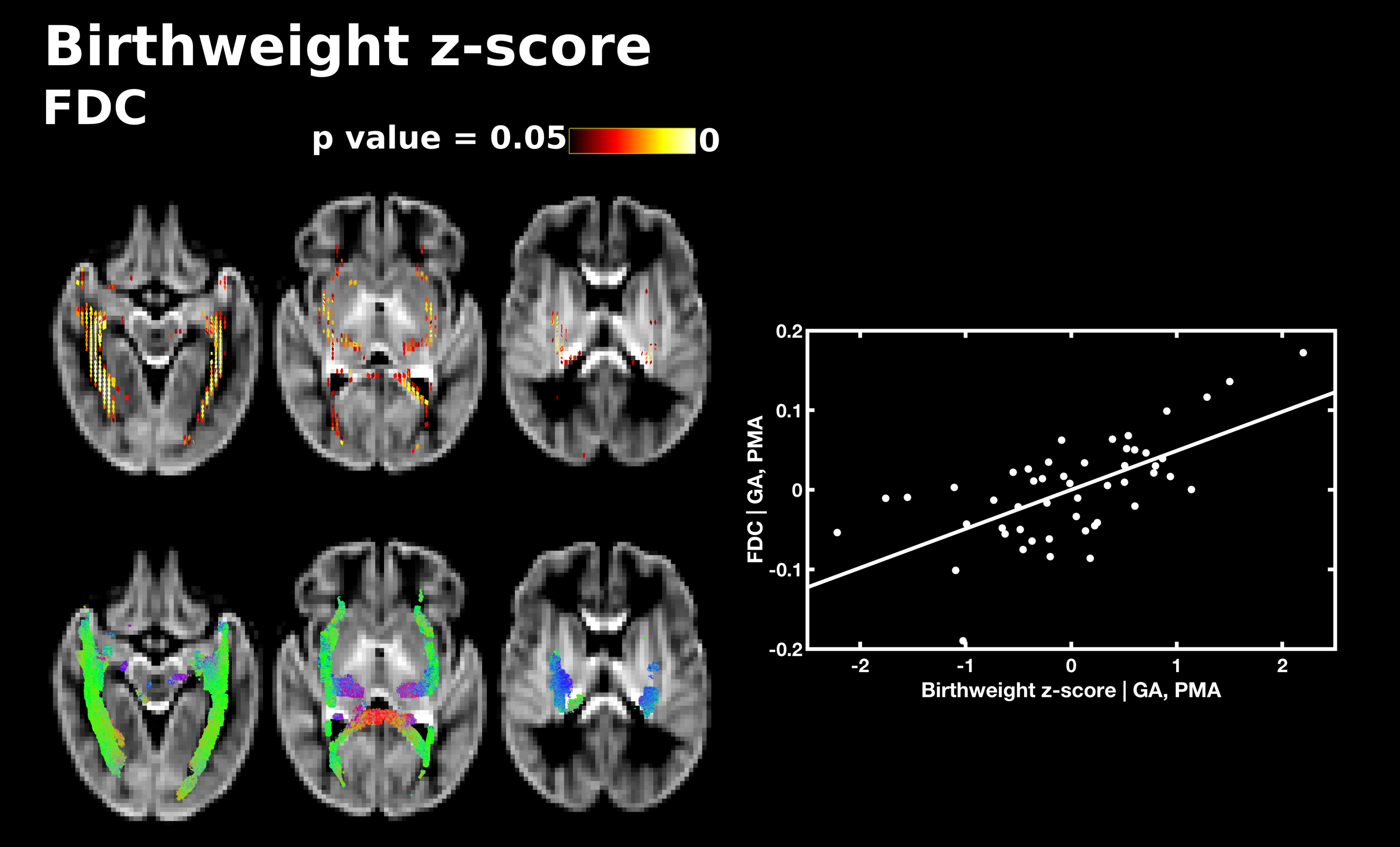  Supplementary Figure 3. The relationship between birthweight z-scores and fibre density and cross-section (FDC), corrected for PMA at scan and GA at birth. Fixels with a significant positive correlation (corrected p < 0.05) are shown on the top row, and streamlines passing through significant fixels (coloured by direction red: left-right; green: anterior-posterior; blue: inferior-superior) are shown on the bottom row, in the axial plane. The scatter plot shows the partial correlation between birthweight z-scores and FDC averaged over all significant fixels, corrected for PMA and GA. |
| --- |

| 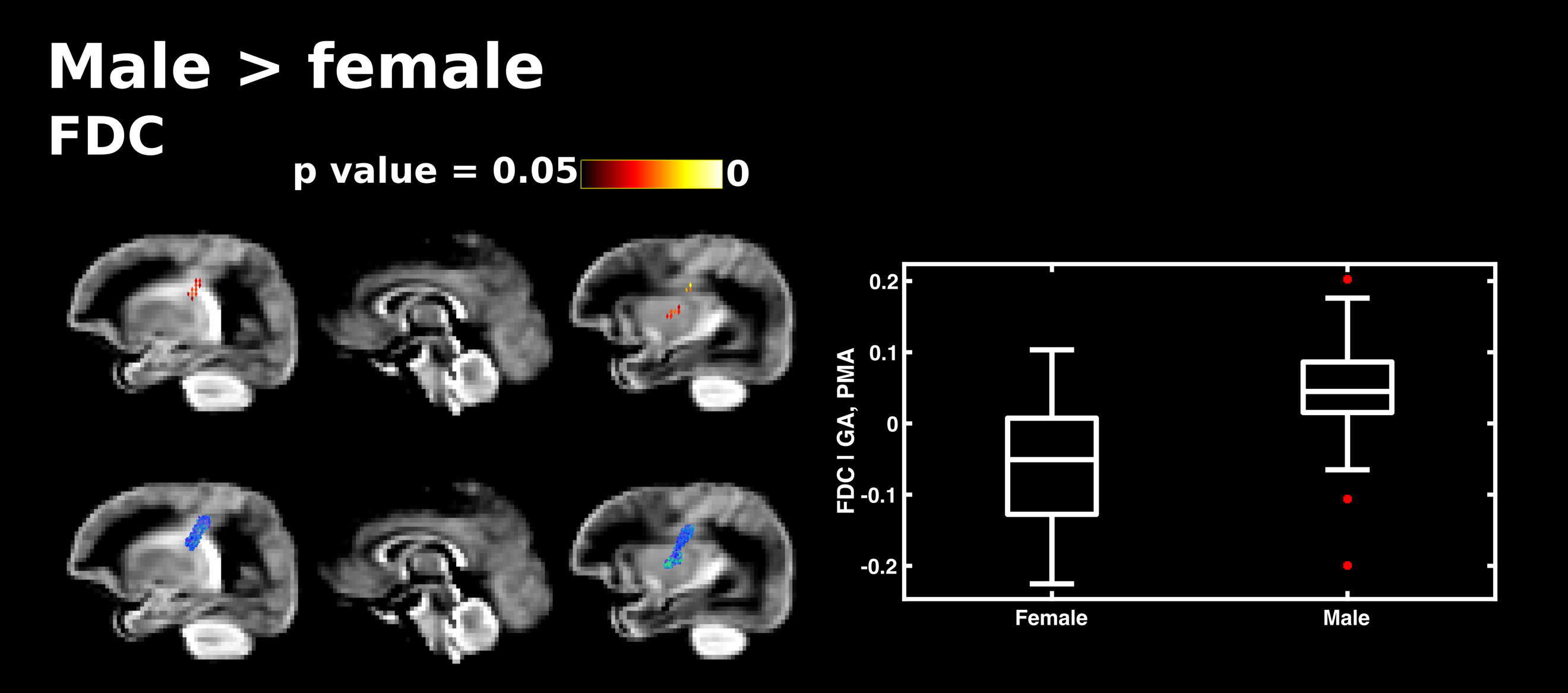  Supplementary Figure 4. Differences in fibre density cross-section (FDC) between male and female subjects, corrected for PMA at scan and GA at birth. Fixels with significantly higher FDC in male subjects (corrected p < 0.05) are shown on the top and streamlines passing through significant fixels (coloured by direction red: left-right; green: anterior-posterior; blue: inferior-superior) are shown on the bottom row in the sagittal plane. The boxplot shows FDC values for male and female subjects averaged over all significant fixels, corrected for GA and PMA. |
| --- |

| 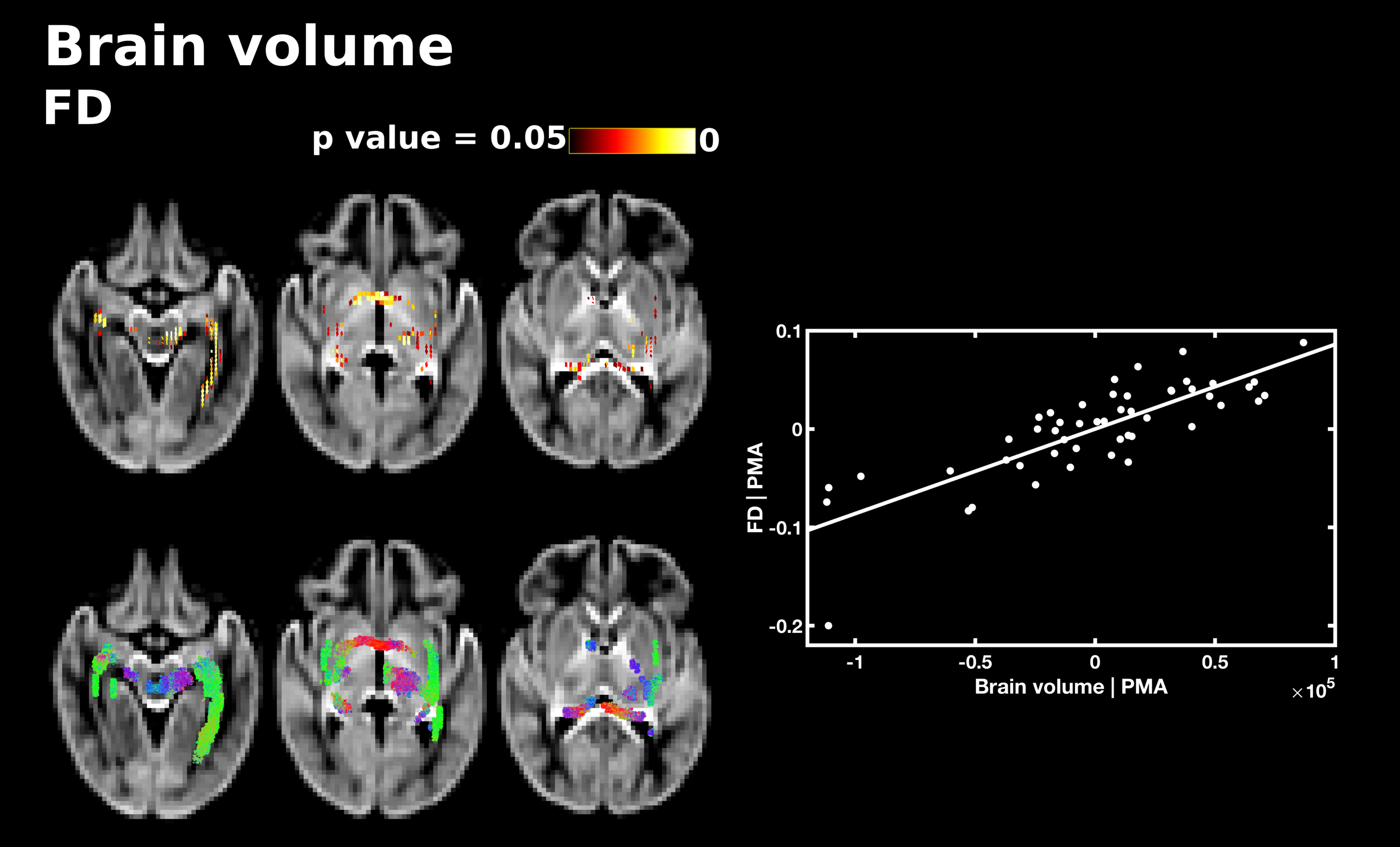  Supplementary Figure 5. The relationship between brain volume at scan and apparent fibre density (FD), corrected for PMA at scan. Fixels with a significant positive correlation (corrected p < 0.05) are shown on the top row, and streamlines passing through significant fixels (coloured by direction red: left-right; green: anterior-posterior; blue: inferior-superior) are shown on the bottom row, in the axial plane. The scatter plot shows the partial correlation between brain volume and FD averaged over all significant fixels, corrected for PMA. |
| --- |

| 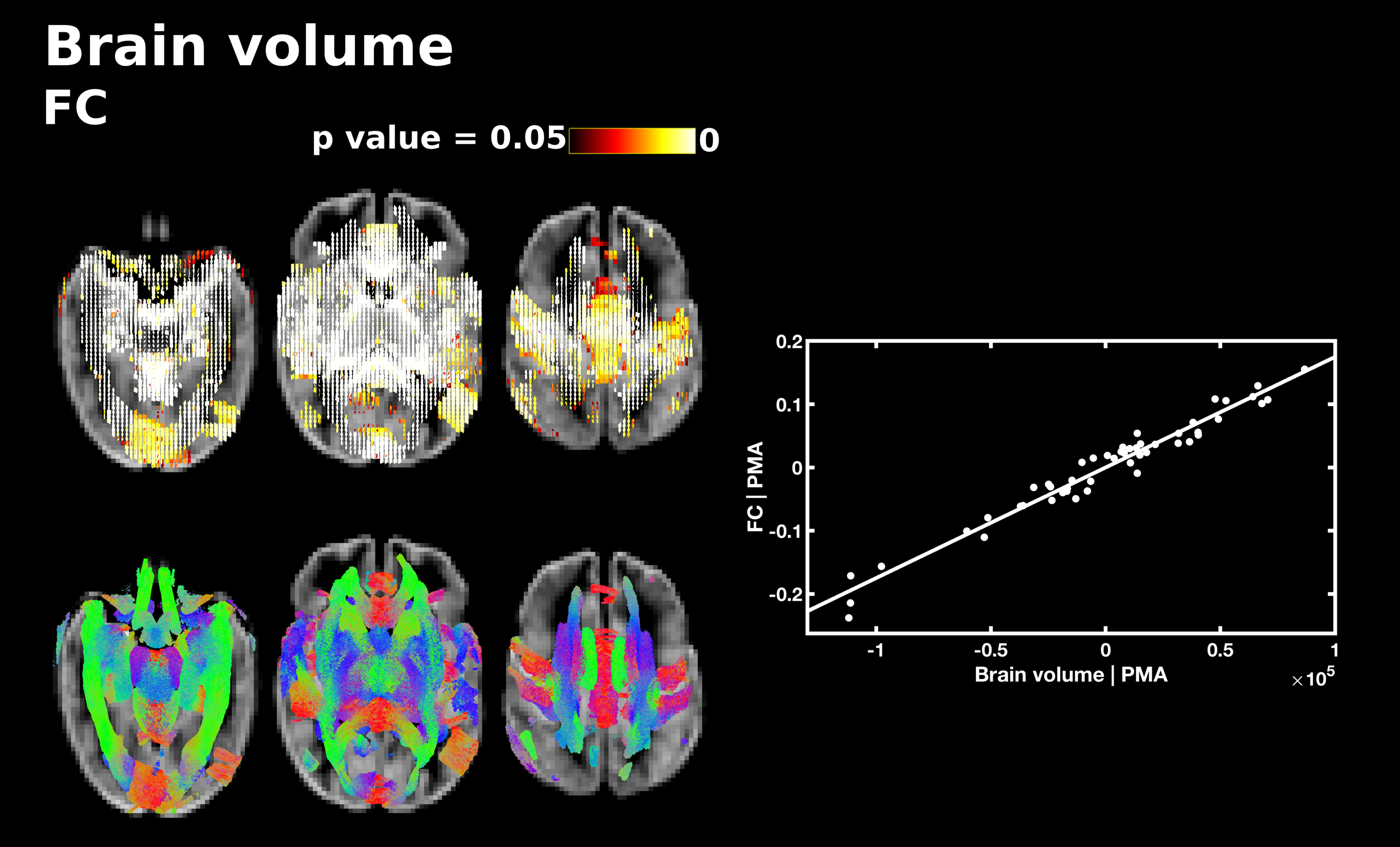  Supplementary Figure 6. The relationship between brain volume at scan and apparent fibre cross-section (FC), corrected for PMA at scan. Fixels with a significant positive correlation (corrected p < 0.05) are shown on the top row, and streamlines passing through significant fixels (coloured by direction red: left-right; green: anterior-posterior; blue: inferior-superior) are shown on the bottom row, in the axial plane. The scatter plot shows the partial correlation between brain volume and FC averaged over all significant fixels, corrected for PMA. |
| --- |

| 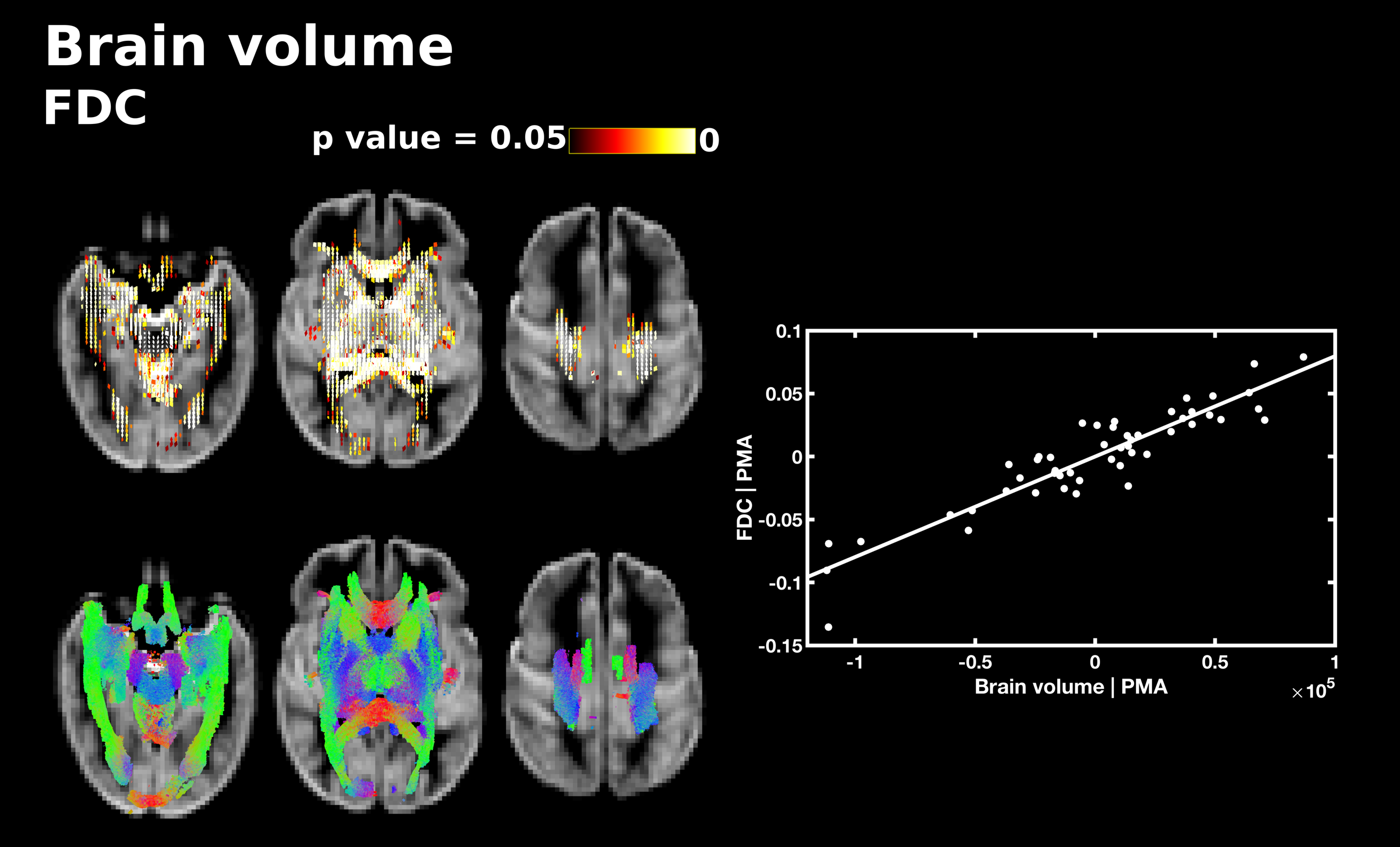  Supplementary Figure 7. The relationship between brain volume at scan and apparent fibre density and cross-section (FDC), corrected for PMA at scan. Fixels with a significant positive correlation (corrected p < 0.05) are shown on the top row, and streamlines passing through significant fixels (coloured by direction red: left-right; green: anterior-posterior; blue: inferior-superior) are shown on the bottom row, in the axial plane. The scatter plot shows the partial correlation between brain volume and FDC averaged over all significant fixels, corrected for PMA. |
| --- |

| 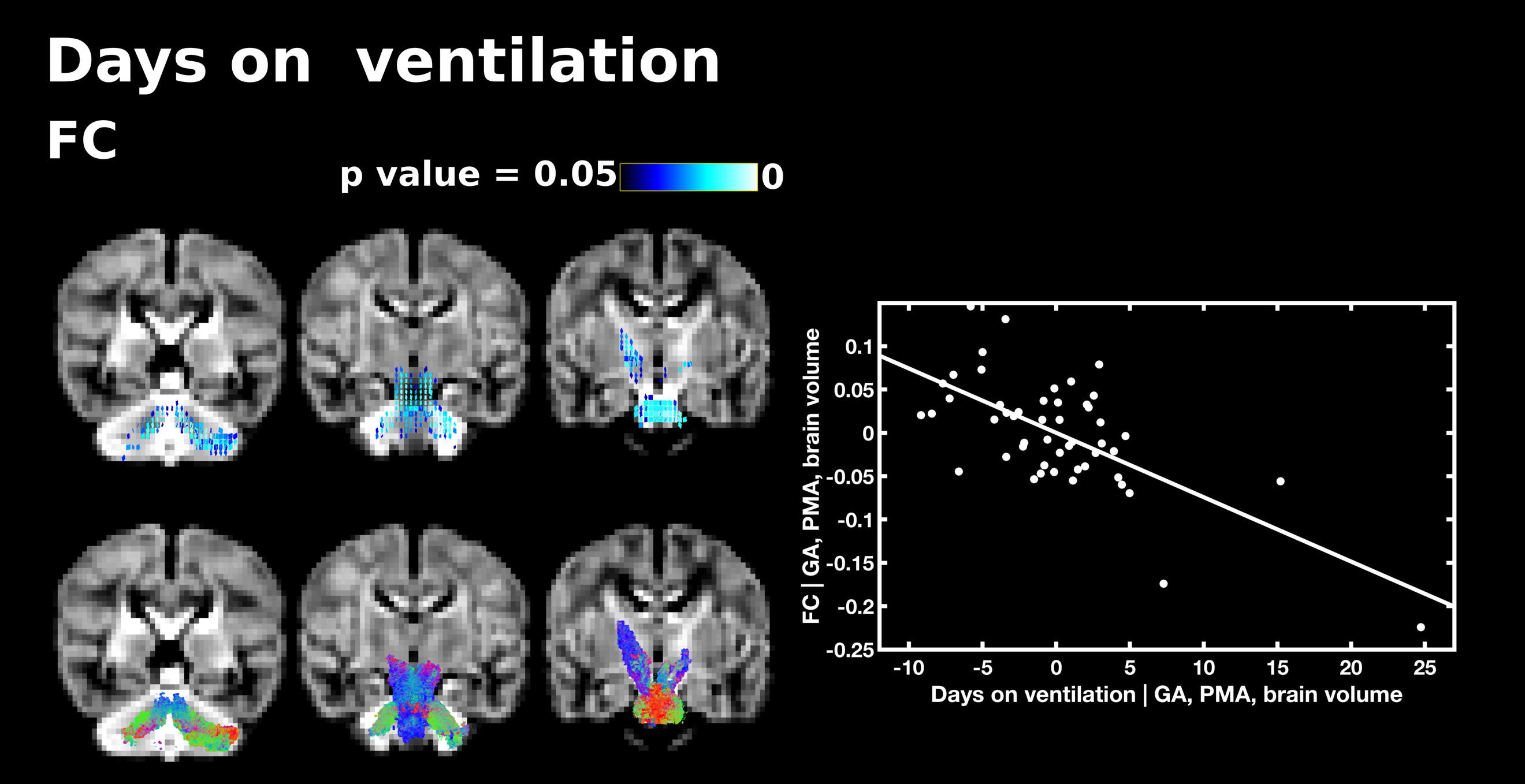  Supplementary Figure 8. The relationship between the number of days requiring mechanical ventilation and fibre cross-section (FC), corrected for PMA at scan, GA at birth and brain volume. Fixels with a significant negative correlation (corrected p < 0.05) are shown on the top row, and streamlines passing through significant fixels (coloured by direction red: left-right; green: anterior-posterior; blue: inferior-superior) are shown on the bottom row, in the coronal plane. The scatter plot shows the partial correlation between days on mechanical ventilation and FC averaged over all significant fixels, corrected for PMA, GA and brain volume. |
| --- |

| 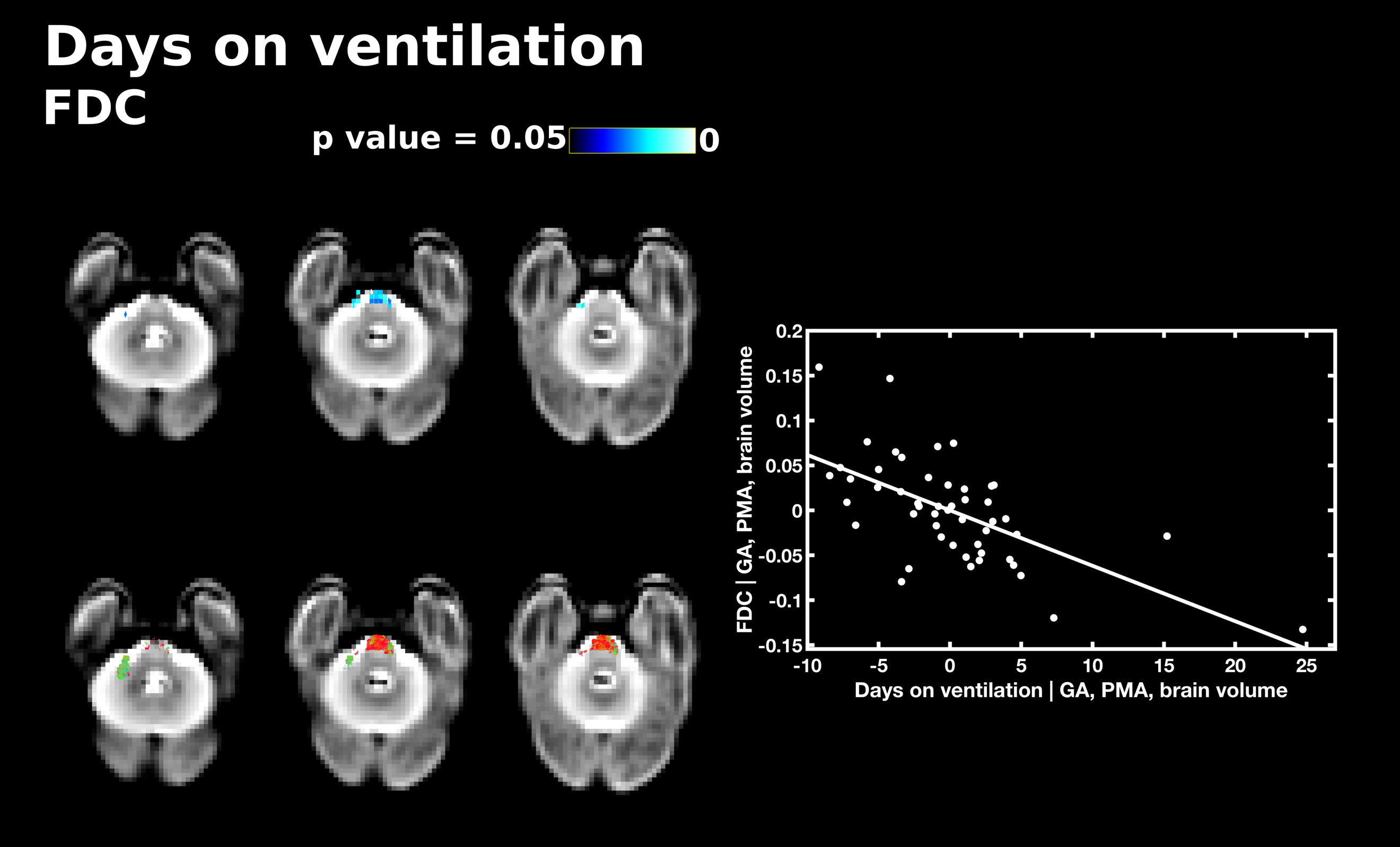  Supplementary Figure 9. The relationship between the number of days requiring mechanical ventilation and fibre density and cross-section (FDC), corrected for PMA at scan, GA at birth and brain volume. Fixels with a significant negative correlation (corrected p < 0.05) are shown on the top row, and streamlines passing through significant fixels (coloured by direction red: left-right; green: anterior-posterior; blue: inferior-superior) are shown on the bottom row, in the coronal plane. The scatter plot shows the partial correlation between days on mechanical ventilation and FDC averaged over all significant fixels, corrected for PMA, GA and brain volume. |
| --- |

Supplementary Table 2. Summary of results from fixel-based analysis. All significant results are bilateral, unless the hemisphere is explicitly stated.

| Variable of interest | FD | FC | FDC |
| --- | --- | --- | --- |
| Brain volume  (corrected for PMA; positive correlation) | Splenium CC  Anterior commissure  L CST  L ILF  R Fornix | Whole white matter | Whole white matter |
| Days on ventilation  (corrected for PMA, GA and total brain volume; negative correlation) | NS | CST  Cerebellum  Pons | Pons |
